# The human ion channel TRPM2 modulates cell survival in neuroblastoma through E2F1 and FOXM1

**DOI:** 10.1038/s41598-022-10385-8

**Published:** 2022-04-15

**Authors:** Iwona Hirschler-Laszkiewicz, Fernanda Festa, Suming Huang, George-Lucian Moldovan, Claudia Nicolae, Ashna Dhoonmoon, Lei Bao, Kerry Keefer, Shu-jen Chen, Hong-Gang Wang, Joseph Y. Cheung, Barbara A. Miller

**Affiliations:** 1 Department of Pediatrics, The Pennsylvania State University College of Medicine, P.O. Box 850, Hershey, PA 17033 USA; 2 Department of Pharmacology, The Pennsylvania State University College of Medicine, P.O. Box 850, Hershey, PA 17033 USA; 3 Department of Biochemistry and Molecular Biology, The Pennsylvania State University College of Medicine, P.O. Box 850, Hershey, PA 17033 USA; 4 Renal Medicine, Brigham and Women’s Hospital, Boston, MA 02115 USA

**Keywords:** Cancer, Cell biology, Oncology

## Abstract

Transient receptor potential channel melastatin 2 (TRPM2) is highly expressed in cancer and has an essential function in preserving viability through maintenance of mitochondrial function and antioxidant response. Here, the role of TRPM2 in cell survival was examined in neuroblastoma cells with TRPM2 deletion with CRISPR technology. Viability was significantly decreased in TRPM2 knockout after doxorubicin treatment. RNA sequence analysis and RT-qPCR revealed reduced RNAs encoding master transcription regulators FOXM1 and E2F1/2 and downstream cell cycle targets including Cyclin B1, CDK1, PLK1, and CKS1. CHIP analysis demonstrated decreased FOXM1 binding to their promoters. Western blotting confirmed decreased expression, and increased expression of CDK inhibitor p21, a CKS1 target. In cells with TRPM2 deletion, cell cycle progression to S and G2/M phases was reduced after treatment with doxorubicin. RNA sequencing also identified decreased DNA repair proteins in cells with TRPM2 deletion after doxorubicin treatment, and DNA damage was increased. Wild type TRPM2, but not Ca^2+^-impermeable mutant E960D, restored live cell number and reconstituted expression of E2F1, FOXM1, and cell cycle/DNA repair proteins. FOXM1 expression alone restored viability. TRPM2 is a potential therapeutic target to reduce tumor proliferation and increase doxorubicin sensitivity through modulation of FOXM1, E2F1, and cell cycle/DNA repair proteins.

## Introduction

Transient receptor potential (TRP) channels are members of a superfamily of cation-permeable ion channels involved in many physiological processes. The melastatin subfamily has a number of members involved in cell proliferation and survival including TRPM1^1,2^, TRPM2^3,4^, TRPM7^5,6^, and TRPM8^7^. TRPM2, the second member of this subfamily to be cloned, is widely expressed in many cell types^4,8^. It is activated by oxidative stress and TNFα^9,10^, through stimulation of production of adenosine diphosphate-ribose (ADPR), which binds to the TRPM2 C-terminal NUDT9-H domain to activate the channel^8,11–14^. TRPM2 is positively regulated by intracellular Ca^2+^ and calmodulin^15,16^, and inhibited by acidification, limiting calcium entry during ischemia^17^. Polymerase poly ADP-ribose (PARP) is involved in activation of TRPM2 through generation of ADPR and the role of PARP inhibitors, which may also modulate TRPM2 function in neurological diseases and in cancer, is under investigation^18,19^.

TRPM2 is highly expressed in many cancers including breast, lung, pancreas, prostate^4,20^, neuroblastoma^21^, and leukemia^3^, suggesting that TRPM2 may promote cell proliferation and/or survival. Targeting the TRPM2 channel promotes cell death in a number of malignancies including T cell leukemia^22^, gastric cancer^23^, and triple-negative and estrogen-receptor positive breast cancer cell lines^4,24^. Work from our laboratory demonstrated that in neuroblastoma and myeloid leukemia, inhibition or deletion of TRPM2 significantly reduces proliferation and increases sensitivity to doxorubicin^3,21,25^. Inhibition of TRPM2 results in mitochondrial dysfunction, decreased bioenergetics through impaired ATP production, increased oxidant stress, and reduced autophagy. We demonstrated that these changes are mediated through decreases in key transcription factors including cAMP-responsive element binding protein (CREB) (which regulates mitochondrial calcium uniporter (MCU) expression and impacts mitochondrial function)^3,26–31^, hypoxia-inducible factor1/2 (HIF-1/2α)^21,25^, Nrf2 (which modulates the antioxidant response)^32^, and ATF4 (which regulates autophagy)^3^. In particular, ROS are significantly increased in cells with TRPM2 deletion as a result of both enhanced ROS production by electron transport chain dysfunction in mitochondria^3,21,25^ and reduced antioxidant response modulated by decreased expression of Nrf2, CREB, and HIF-1α^32^. This would contribute to increased sensitivity to doxorubicin treatment, which itself induces oxidative stress, by increasing ROS above a cytotoxic threshold^25,33,34^.

In neuroblastoma, cell proliferation is reduced following deletion of TRPM2, suggesting that TRPM2 may be involved in modulation of cell cycle, which is explored here. Cell cycle proteins are expressed in two major waves, one during DNA synthesis (S phase) and a second during mitosis (M phase). Expression of the majority of cell cycle genes is highly regulated at the transcriptional level by repressor retinoblastoma (RB) pocket proteins (RB, p107, p130), activator E2F (adenovirus early gene 2 binding factor) transcription factor family, and MuvB (multi-vulva class B) core complex containing LIN52, LIN9, LIN54,and LIN37^35^. G1/S genes are repressed by interaction of RB-E2F complexes or Rb pocket proteins and DREAM (dimerization partner **D**P, **R**B-like proteins, **E**2F, **A**nd **M**uvB) complexes with E2F promoter sites. G1/S genes are expressed through binding of activating E2F/DP complexes, when they are released from RB pocket proteins following pocket protein phosphorylation by cyclin-dependent kinases^35^. E2F1-3 transcription factors are then available to promote expression of genes controlling cell cycle S-phase entry, DNA-damage response, and mitosis^36,37^. G2/M genes are similarly repressed by binding of RB-like pocket proteins to DREAM complexes at specific sites, and activated by sequential binding of B-MYB-MuvB and FOXM1-MuvB complexes following phosphorylation and release of RB-like pocket proteins^35^. FOXM1 is a master regulator of transcription and functions in cell proliferation, cell cycle progression, DNA repair, and tumorigenesis^38^. FOXM1 sustains proliferation by modulating expression of a number of cell cycle proteins^39^. Its overexpression in many human cancers frequently indicates a poor prognosis and is associated with chemotherapy resistance^38,39^. In contrast, reduced expression or inhibition of FOXM1 results in decreased proliferation, migration, metastasis, and epithelial to mesenchymal transition^38^. E2F transcription factors, CREB, and HIF-1/2α all promote FOXM1 transcription and expression^38,39^. E2Fs, particularly E2F1, also function as master regulators in cancer and regulate a number of pathways which promote proliferation^40^.

High ROS levels in cells with TRPM2 deletion can damage DNA. When DNA is damaged, pathways including activation of the DNA checkpoint at G2/M can halt cell cycle to allow more time for DNA repair or result in apoptosis if DNA is extensively damaged. FOXM1 mediates transcription of genes regulating DNA damage sensing and DNA repair^41^. E2F1 also has roles in DNA damage response independent of transcription through mechanisms including promotion of nucleotide excision repair of UV-induced DNA damage and recruitment of repair factors to sites of DNA damage^42,43^.

Because TRPM2 deletion results in reduced cell proliferation, survival after doxorubicin treatment, and increased ROS levels which damage DNA, here we investigated the role of TRPM2 in regulation of cell cycle and DNA repair in neuroblastoma cells. Live cell number and viability were significantly decreased in TRPM2 KO cells compared to control after doxorubicin treatment. Major findings are: (1) the master transcription factor FOXM1 is reduced in TRPM2 deletion, as are transcription factors which regulate FOXM1 expression including E2F1; (2) expression of downstream targets of FOXM1 which modulate the cell cycle including cyclin B1, cyclin-dependent kinase 1 (CDK1), polo-like kinase 1 (PLK1), and cyclin-dependent kinase regulatory subunit 1 (CKS1) are decreased in TRPM2 KO; (3) inhibition of TRPM2 function significantly alters cell cycle progression, increasing the percentages of cells in Sub-G0 and G0/G1 phases of the cell cycle and reducing the percentages of cells in S and G2M after doxorubicin; (4) DNA damage is significantly increased and DNA repair proteins are also decreased in cells with TRPM2 deletion after doxorubicin treatment; and (5) reconstitution of TRPM2 with wild type TRPM2 but not Ca^2+-^impermeable E960D mutant restores cell proliferation, viability, expression of cell cycle and DNA repair proteins, and reduces doxorubicin sensitivity, demonstrating an important role for TRPM2-mediated calcium influx. Determination of the role of TRPM2 in doxorubicin sensitivity of the myeloid leukemia cell line U937 similarly demonstrated reduced FOXM1, E2F1, and expression of proteins involved in cell cycle and DNA damage response in cells with TRPM2 deletion.

## Results

### TRPM2 KO in neuroblastoma reduces cell proliferation and increases doxorubicin sensitivity

**Figure 1:**
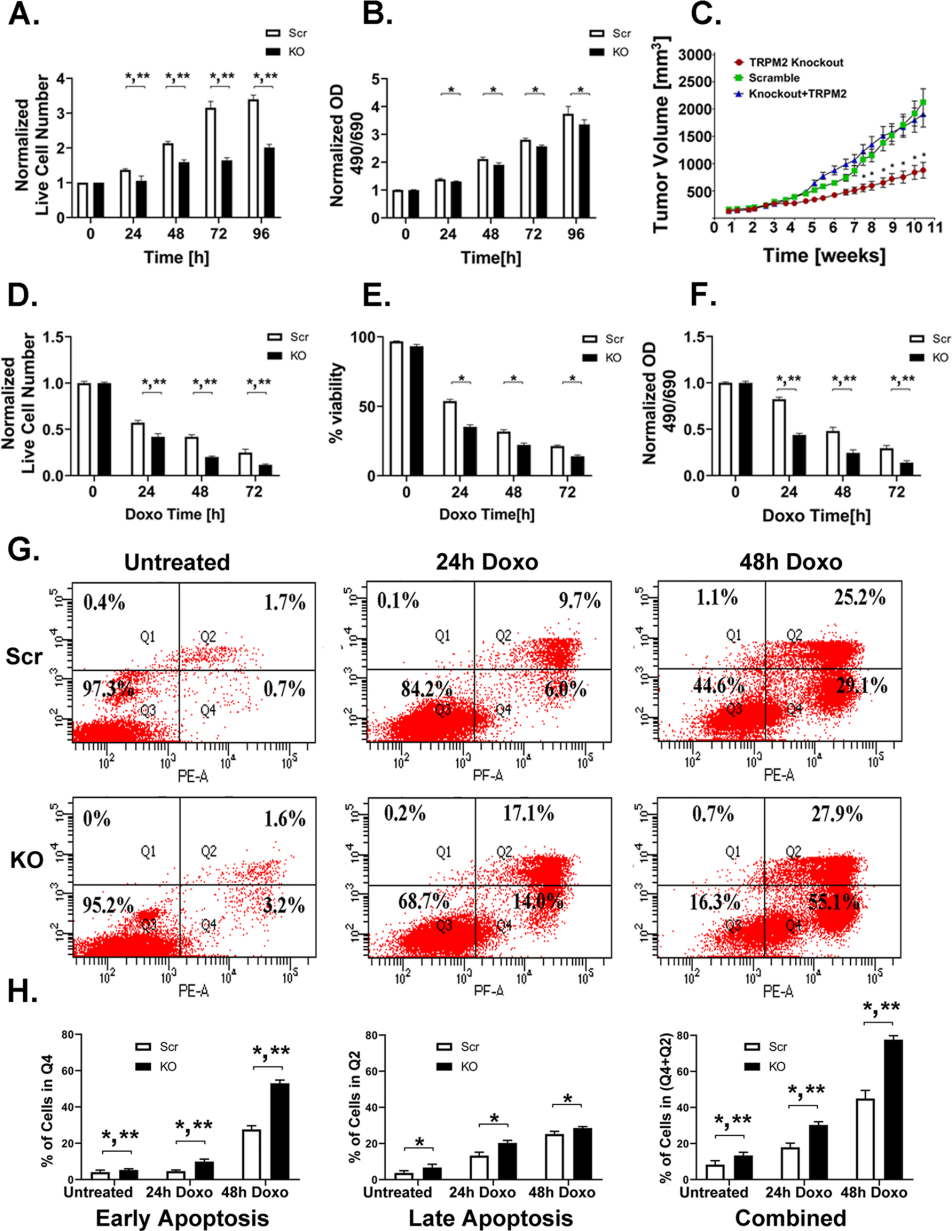
TRPM2 KO in neuroblastoma reduces proliferation, viability and increases apoptosis. Cell proliferation and viability were studied in SH-SY5Y cells in which TRPM2 was deleted with CRISPR technology (KO) and in scrambled control (Scr). (**A**, **B**) Cell proliferation was measured in two clones of TRPM2 deleted (KO) and in scrambled control (Scr) SH-SY5Y cells with trypan blue exclusion (**A**) and XTT analysis (**B**), 24 to 96 h after plating in two experiments. Mean ± S.E.M. is shown (**A**, n = 16/group; **B**, n = 12/group). (**C**) Xenograft tumor growth was measured in tumors from two clones of KO, scrambled, or KO cells reconstituted with wild type TRPM2. Mean ± S.E.M. for two clones combined (n = 21/group). (**D**–**F**) Cell viability was measured with trypan blue (**D**, **E**) and XTT analysis (**F**) 24 to 72 h after treatment with 0.3 µM doxorubicin in two experiments (**D**, **E**, n = 16/group; F, n = 12/group). Results were normalized to time 0 and mean ± S.E.M. from two experiments is shown. (**G**, **H**) Apoptosis was measured in Scr and TRPM2 KO synchronized cells untreated or treated with doxorubicin (0.3 µM) for 24 or 48 h. (**G**) Cytometric analysis in a representative experiment is shown. (**H**) Mean ± S.E.M. percent of early apoptotic (Q4), late apoptotic (Q2) and all apoptotic (Q2 + Q4) cells in three experiments including the one shown in G (n = 5 replicates untreated cells; n = 8, 24 h; n = 6, 48 h). Statistics (**A**–**H**) two-way ANOVA, *p < 0.01, indicates significant differences between Scr vs KO, Group Effect; **p < 0.0001, group x time (indicates the differences between the groups significantly increased with time, **A**) or group × doxorubicin exposure time interaction effect (indicates the differences between groups significantly increased with time of doxorubicin exposure, **D**, **F**, **H**).

TRPM2 is highly expressed in neuroblastoma^21^. SH-SY5Y neuroblastoma cells in which TRPM2 was deleted with CRISPR technology demonstrated significantly reduced proliferation compared to scrambled control cells, quantitated with trypan blue exclusion (Fig. 1A) or XTT analysis (Fig. 1B). To further examine the role of TRPM2 in in vivo neuroblastoma tumor growth, athymic female mice were injected subcutaneously with 1.5 × 10^7^ SH-SY5Y cells in which TRPM2 was deleted, scrambled control cells, or cells with TRPM2 deletion in which TRPM2 was reconstituted by stable transfection of full length wild type channel. Tumors from cells with TRPM2 deletion demonstrated significantly reduced volume compared to scrambled controls (Fig. 1C). Tumor growth of cells with TRPM2 deletion was restored to that of control cells by TRPM2 reconstitution, demonstrating absence of significant secondary off-target effects in KO cells.

To determine the effect of TRPM2 deletion on doxorubicin sensitivity, cells with TRPM2 deletion or control scrambled cells in culture were treated with doxorubicin for 24, 48, or 72 h. After treatment with doxorubicin, live cell number measured with trypan blue exclusion (Fig. 1D) or XTT analysis (Fig. 1F) and percent viability (Fig. 1E) were significantly reduced in TRPM2 KO cells compared to controls (Figs. 1D–F), and the decrease in live cell number increased significantly with time of doxorubicin exposure, as shown previously^21,25,32^. In addition, TRPM2 KO cells treated with doxorubicin had greater percentages of cells in both early and late stages of apoptosis (Fig. 1G,H). Collectively, our findings demonstrate increased sensitivity of cells with TRPM2 deletion to doxorubicin.

### Expression of the master transcription factors E2F1 and FOXM1 and downstream targets involved in cell cycle progression is reduced in cells with TRPM2 deletion after doxorubicin treatment

To identify mechanisms through which TRPM2 modulates proliferation and doxorubicin sensitivity in neuroblastoma, RNA seq analysis was performed. Our analysis compared mRNA levels in cells with TRPM2 deletion to control cells, which were untreated or treated with doxorubicin for 24 h. The majority of genes with increased or decreased mRNA expression in untreated cells with TRPM2 deletion were also found in doxorubicin treated cells, but additional differences were identified after doxorubicin treatment. The top cell signaling pathways that were identified as different in cells with TRPM2 deletion compared to control cells after doxorubicin treatment are shown in Supplementary Fig. S1. These included RNAs involved in “Cell Cycle:G2/M DNA Damage Checkpoint Regulation”, “Mitotic Roles of Polo-like Kinase”, and “Cyclins and Cell Cycle Regulation.” The overlap of gene differences between TRPM2 deletion versus control in untreated cells and doxorubicin treated cells is shown in Supplementary Fig. S2. The RNA seq data discussed in this publication have been deposited in NCBI’s Gene Expression Omnibus^44,45^ and are accessible through GEO Series accession number GSE197243.

**Figure 2:**
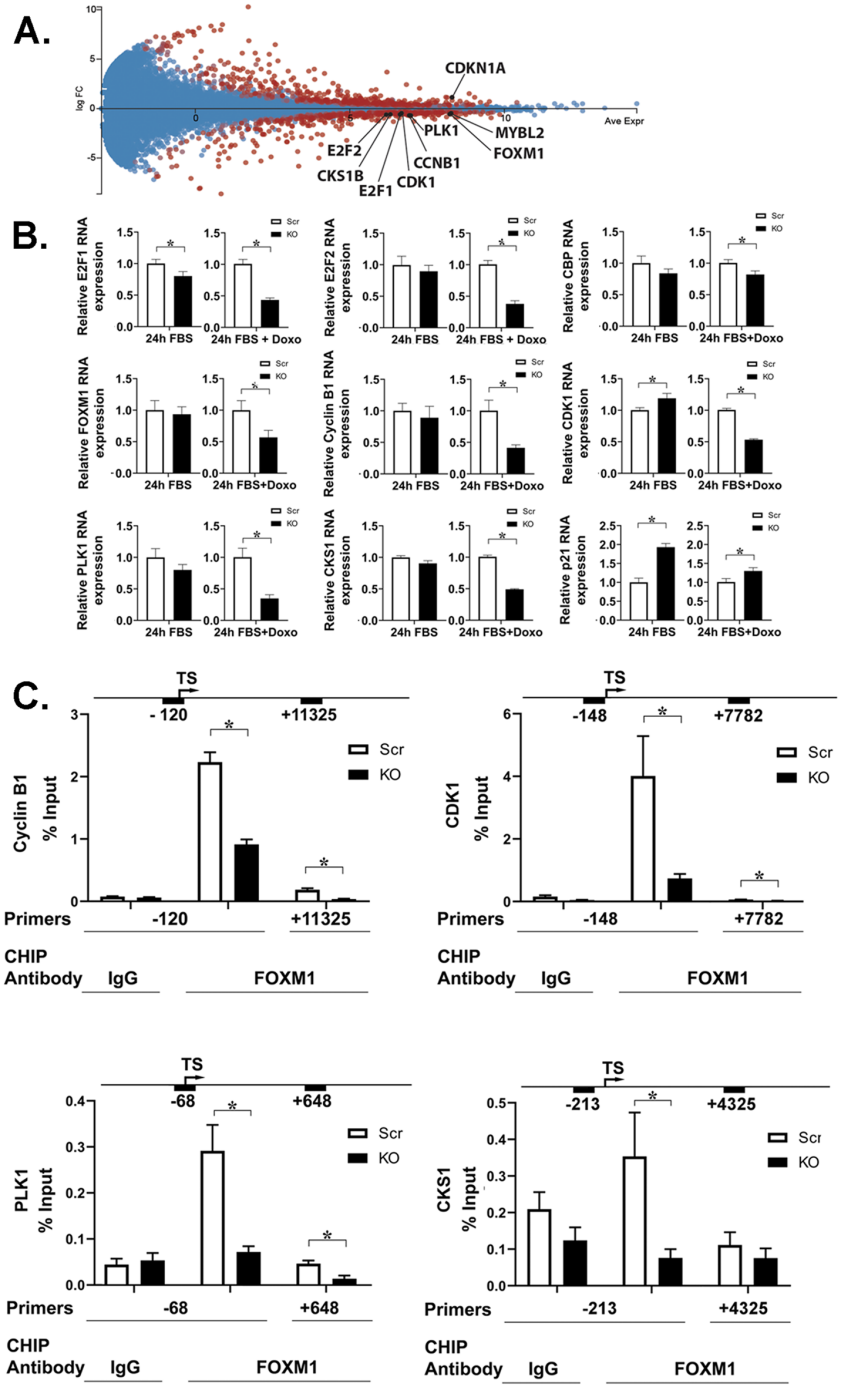
RNA analysis of transcription factors regulating cell cycle genes and downstream targets in TRPM2 KO. (**A**) RNA seq of cell cycle gene expression. MA plot (log ratio vs abundance) of RNA seq data comparing SH-SY5Y scrambled control (Scr) and TRPM2 deleted (KO) cells after 24 h treatment with doxorubicin. Two biological replicas of two clones are combined. Genes with q-value < 0.05 are displayed in red and relevant cell cycle genes are labeled. Log FC < 0 indicates Scr > KO. Figure was prepared using the Degust 4.1.1 web-tool for RNA seq analysis (https://degust.erc.monash.edu/). (**B**) RT-qPCR of E2F1, E2F2, CBP, FOXM1, Cyclin B1, CDK1, PLK1, CSK1, and p21 RNA from TRPM2 KO or Scr SH-SY5Y cells. Amount in KO was normalized to Scr for each experimental group. Mean ± S.E.M. of three experiments, each performed in triplicate (n = 9), is shown. Statistics: Student’s t test, *p < 0.05, Scr vs KO. (**C**) CHIP analysis of FOXM1 binding to Cyclin B1, CDK1, PLK1 and CKS1 promoters was performed with KO or Scr cells using control IgG or anti-FOXM1 antibody as described in Methods. Mean ± S.E.M FOXM1 binding to the promoter as percent of input from two independent experiments with 3 (PLK1) or 4 (Cyclin B1, CDK1, CKS1) replicates in each (n = 6 to 8 per group) is shown. Statistics: unpaired t test, *p ≤ 0.03. Numbers above graph correspond to the start of forward primer.

To identify mechanisms involved in the increased proliferation and reduced doxorubicin sensitivity of neuroblastoma cells with TRPM2 expression compared to cells with TRPM2 deletion, the focus of this manuscript, we first examined genes involved in cell cycle progression. In cells with TRPM2 deletion treated with doxorubicin, RNA seq showed significantly reduced mRNA for the master transcription factors E2F1, E2F2, and FOXM1 (Fig. 2A). In addition, genes involved in cell cycle modulation and downstream of FOXM1 that were downregulated in TRPM2 deletion were identified including Cyclin B1 (CCNB1), CDK1, PLK1, and CKS1, (Fig. 2A), and mRNA for cyclin dependent kinase inhibitor CDKN1A (p21) was increased. RT-qPCR confirmed RNA seq results, demonstrating a role for TRPM2 in transcriptional regulation of these genes, particularly after doxorubicin treatment (Fig. 2B). RNA seq data did not identify a difference in expression levels of CREB binding protein (CBP), a key FOXM1 regulator and co-activator. However, because of its important role as a transcriptional coactivator, RT-qPCR was performed, which showed that CBP expression was significantly decreased in cells with TRPM2 deletion after doxorubicin treatment. These changes with RT-qRCR reached statistical significance in all groups after doxorubicin treatment, but only E2F1, CDK1, and p21 were statistically different in untreated cells with TRPM2 deletion compared to control.

CHIP analysis demonstrated that binding of FOXM1 to the Cyclin B1, CDK1, PLK1, and CKS1 promoters was significantly reduced in cells with TRPM2 deletion after doxorubicin treatment (Fig. 2C), indicating that decreased levels of FOXM1 are involved in the reduced expression of these cell cycle genes.

**Figure 3:**
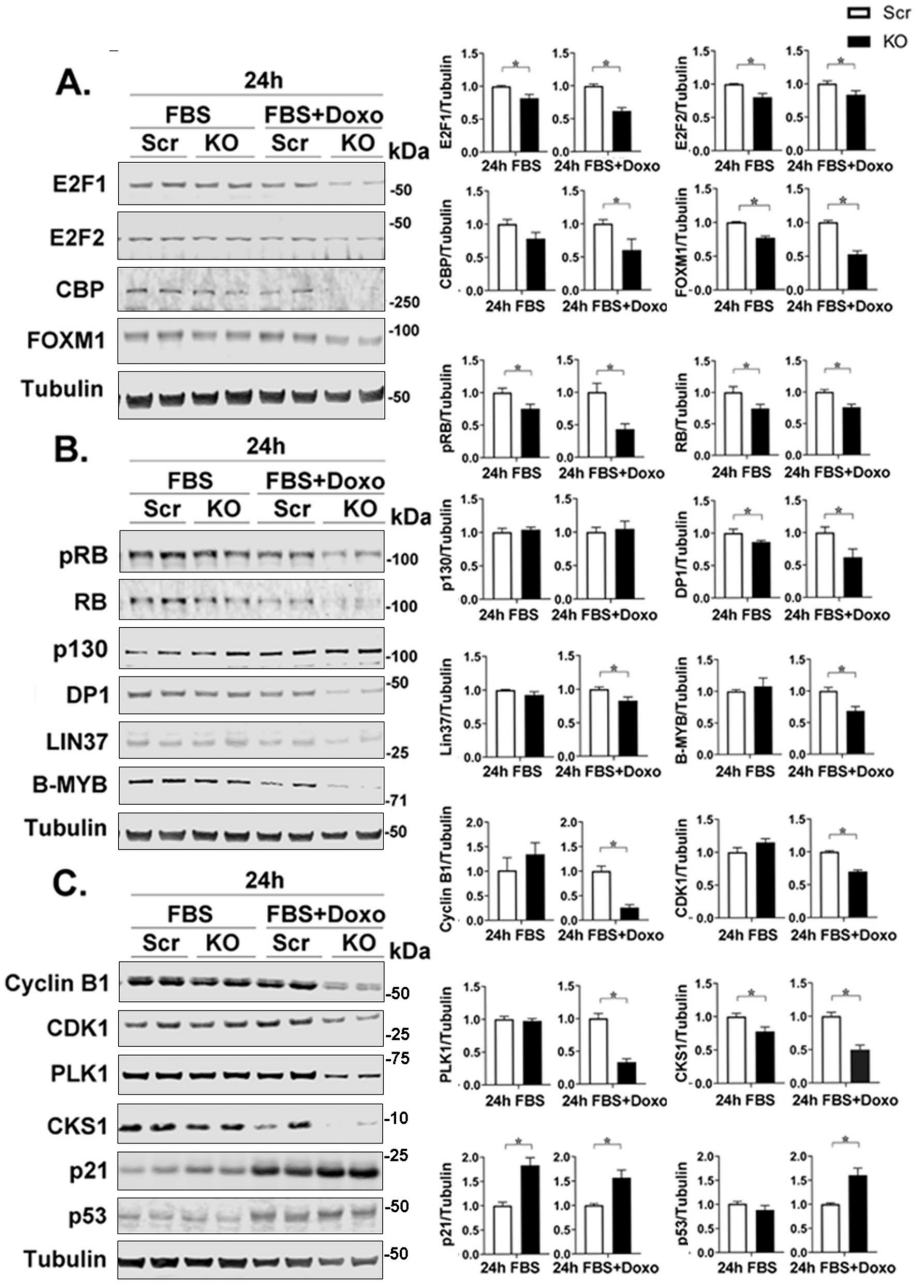
TRPM2 KO reduces expression of E2F1/2, FOXM1, and DREAM complex and cell cycle proteins. Western blotting was performed on two clones of TRPM2 KO and scrambled SH-SY5Y cells after serum deprivation followed by refeeding with 10% FBS with or without 0.3 µM doxorubicin for 24 h. Representative Western blots demonstrate expression of (**A**) transcription factors E2F1, E2F2, CBP, FOXM1, and tubulin control; (**B**) regulatory proteins pRB (pS795), RB, RB-like pocket protein p130, DP1, DREAM/MuvB complex member LIN37, and B-MYB; (**C**) downstream transcriptional targets of FOXM1 including Cyclin B1, CDK1, PLK1, CKS1, p21 and p53. Samples (Scr, KO) for each blot were derived from the same experiment and always processed in parallel. The different probes for each antibody are made explicit with white space and delineated with a black box. Densitometry measurements from three experiments for each protein were standardized to tubulin and to each experiment’s scrambled control. Means ± S.E.M. for each group are shown on the right. Statistics: unpaired t test, *p < 0.05. Full length gels for Western blots are shown in Supplementary Fig. S3.

Western blotting confirmed downregulation of the master transcription regulator proteins FOXM1 and E2F1/E2F2 in cells with TRPM2 deletion. These proteins were reduced in both untreated and cells treated with doxorubicin (Fig. 3A). The decrease in E2F2, and FOXM1 protein found in untreated cells with TRPM2 deletion was not detected at the mRNA level with RT-qPCR, suggesting it may be mediated by translational/posttranslational mechanisms. FOXM1 transcription is positively regulated by factors including E2F1/2, CREB, CBP, and HIF-1α, which are decreased in cells with TRPM2 deletion^21,25,26^ and may contribute directly to reduced FOXM1 expression. CBP was also found to be decreased in cells with TRPM2 deletion after doxorubicin treatment with Western blotting (Fig. 3A), and as a transcriptional coactivator of both E2F1^46^ and FOXM1^47^, may contribute to reduced transcription of FOXM1 and its downstream cell cycle target genes.

Modulation of other proteins in the DREAM/MuvB and RB/E2F complexes, in addition to FOXM1, which are involved in cell cycle transcriptional control by TRPM2 were examined^35^. The DREAM/MuvB complex can act as a repressor of cell cycle in G1 and S phases, and a repressor or activator in G2 and M^35^. When RB and RB-like pocket proteins are phosphorylated by cyclin-CDK complexes, they no longer bind E2F and DP1 sites, and transcription of G1/S genes can proceed. Here, in cells with TRPM2 deletion, the quantity of negative regulator RB and pRB were reduced (Fig. 3B). Negative regulator RB-like pocket proteins p130 and p107 (not shown) were not different in the KO, but positive regulators E2F1/2 and DP1 were decreased in untreated and doxorubicin treated cells with TRPM2 deletion (Fig. 3A,B). To facilitate expression of G2/M genes, FOXM1 is recruited by the activator MMB (B-MYB-MuvB) complex. Expression of key members of the Dream/MuvB complex required for DREAM function were decreased in TRPM2 deletion including FOXM1, LIN37, and the positive regulator B-MYB after doxorubicin treatment (Fig. 3B).

Expression of key transcriptional targets of FOXM1 involved in facilitating cell cycle progression was examined at the protein level including Cyclin B1, CDK1, PLK1, and CKS1^48,49^. Cyclin B1 is expressed in G2/M phase of the cell cycle and is phosphorylated by PLK1 and CDK1, resulting in its nuclear relocation and promotion of mitosis. Cyclin B1, CDK1, and PLK1 were all significantly decreased in cells with TRPM2 deletion after doxorubicin treatment (Fig. 3C). CKS1 is involved in ubiquitination and degradation of the cell cycle inhibitor p21^50^. CSK1 was decreased and its target cyclin-dependent kinase inhibitor p21 was increased in both untreated and doxorubicin treated cells (Fig. 3C). Increased expression of p53 expression observed here may contribute to modulation of FOXM1 and p21 expression by TRPM2 because p53 represses FOXM1 transcription and activates p21 expression.

### TRPM2 deletion modulates cell cycle progression after doxorubicin

**Figure 4:**
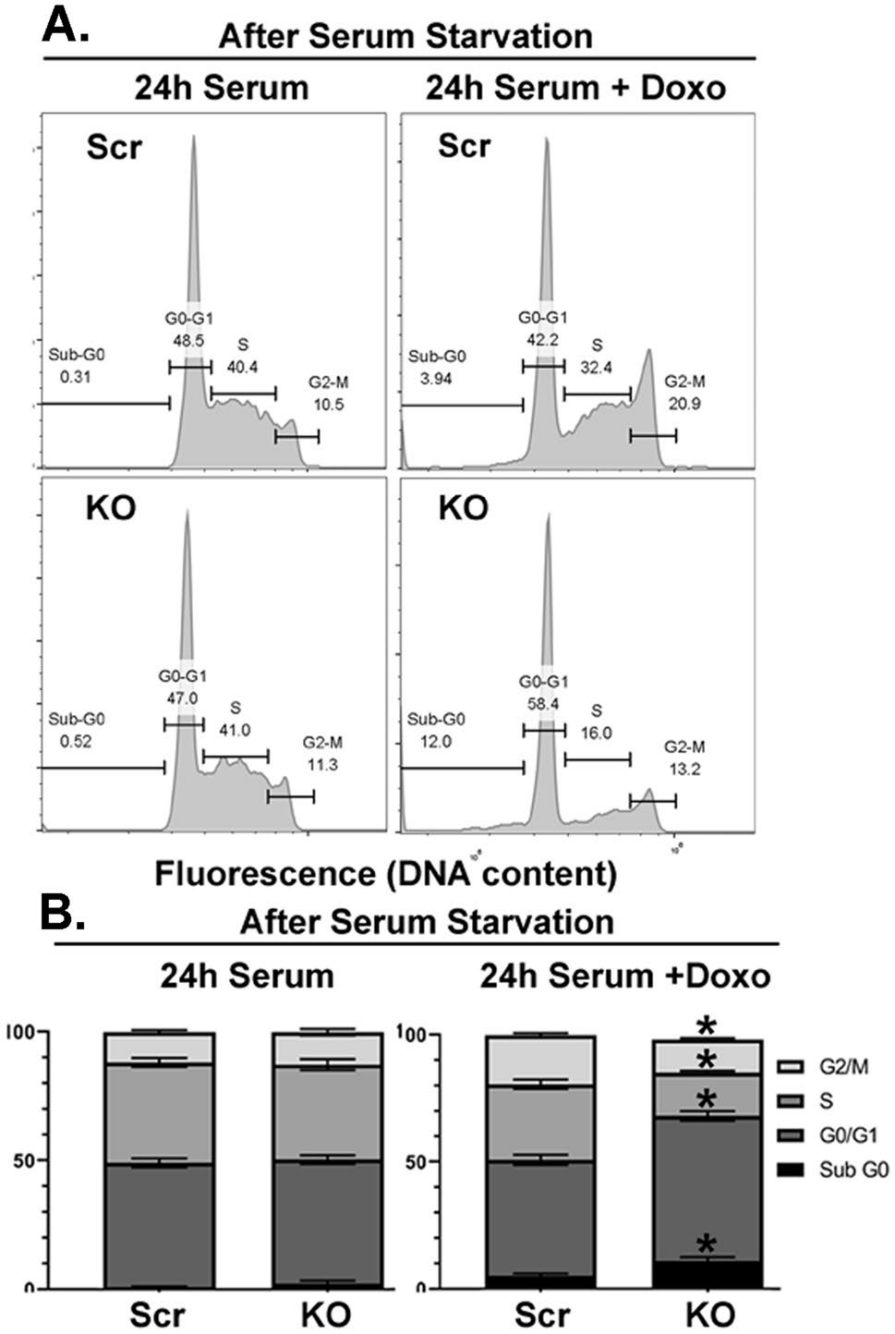
TRPM2 KO alters cell cycle distribution after exposure to doxorubicin. TRPM2 KO or Scr control SH-SY5Y cells were synchronized in culture by 12–18 h of serum deprivation, followed by refeeding for 24 h with 10% FBS with or without 0.3 µM doxorubicin. Percent of cells in different phases of the cell cycle was determined as described in “Methods”. (**A**) Tracing of the number of cells in different phases of the cell cycle in one of six experiments. (**B**) Mean ± S.E.M. percent of cells in each phase of the cell cycle (Sub-G0, G0/G1, S, or G2/M) in six experiments (n = 12/group). Statistics: unpaired, two-tailed t-test, *p < 0.006, Scr vs KO.

Because of reduced expression of cell cycle proteins in cells with TRPM2 deletion, the involvement of TRPM2 in cell cycle progression was examined. The percent of cells in each phase in control cells and cells with TRPM2 deletion cultured with FBS was similar (Fig. 4). After 24 h of doxorubicin exposure, cells with TRPM2 deletion showed significantly greater percentages of cells in Sub-G0 and G0/G1, consistent with the increase in apoptotic cells (Fig. 1G,H), and significantly lower percentage of cells in S and G2/M phases (Fig. 4), consistent with reduced expression of key cell cycle proteins.

### Reconstitution of TRPM2 deletion by wild type TRPM2 but not the Ca^2+^-impermeable E960D mutant restores cell cycle protein expression

**Figure 5:**
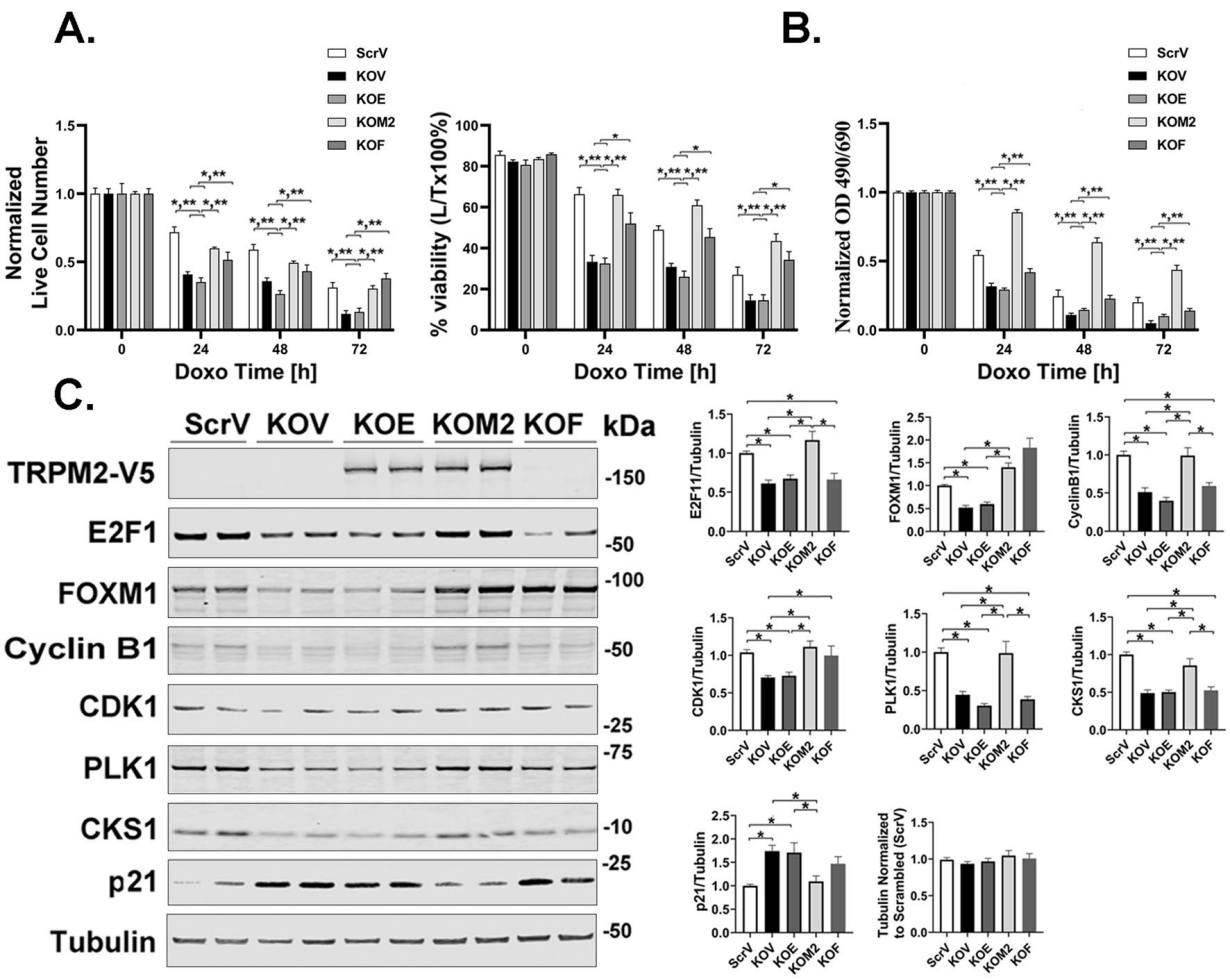
Reconstitution of TRPM2 in KO cells restores viability and expression of cell cycle proteins. TRPM2 KO SH-SY5Y cells were stably transfected with empty vector (KOV), TRPM2 calcium impermeant mutant E960D (KOE), wild type TRPM2 (KOM2), or FOXM1b (KOF). Live cell number and viability were measured after treatment with 0.3 µM doxorubicin for 0–72 h with trypan blue exclusion (**A**) and XTT analysis (**B**). Results were normalized to time 0. Percent viability was calculated as live divided by total cell number × 100%. (**A**, **B**) Mean ± S.E.M for two experiments (n = 12–24). Statistics: two-way ANOVA. *p < 0.0001, indicates significant differences between Scr vs KO, Group Effect; **p < 0.0001, group x doxorubicin exposure time interaction effect (indicates the differences between groups significantly increased with time of doxorubicin exposure). (**C**) Western blotting was performed on lysates from two clones for each group after doxorubicin treatment for 24 h. Blots were probed with antibody to V5 to demonstrate successful transfection of TRPM2 E960D or M2, and antibodies to E2F1, FOXM1, Cyclin B1, CDK1, PLK1, CKS1, p21, and tubulin. Samples (ScrV, KOV, KOE, KOM2, KOF) were derived from the same experiment and always processed in parallel. One blot of four to five experiments is shown. The different probes for each antibody are made explicit with white space and delineated with a black box. Mean ± S.E.M. densitometry measurements of the four to five experiments with 2 clones for each protein in each experiment (n = 8–10) are shown. Measurements were standardized to tubulin and to each experiment’s average scrambled control. Statistics: one-way ANOVA, *p < 0.05. Full length gels for Western blots are shown in Supplementary Fig. S4.

To determine whether calcium influx through TRPM2 is required for modulation of cell cycle protein expression, SH-SY5Y TRPM2 KO cells stably transfected with empty V5 vector (KOV), wild type TRPM2 (KOM2), TRPM2 Ca^2+^-impermeable mutant E960D (KOE)^31,51^, or FOXM1b (KOF), were treated with doxorubicin. Live cell number and viability were significantly reduced in cells with TRPM2 deletion (Figs. 5A,B), and were restored to control by wild type TRPM2 but not the E960D mutant^3,25,26^. Expression of transcription factors E2F1, FOXM1, and downstream targets Cyclin B1, CDK1, PLK1, CKS1, and p21 were also restored by wild type TRPM2 but not E960D (Fig. 5C), demonstrating that calcium influx through TRPM2 was required. FOXM1b expression alone restored cell proliferation and viability after doxorubicin (Fig. 5A,B). However, while FOXM1b alone restored expression of CDK1, it did not restore expression of E2F1, Cyclin B1, PLK1, or CKS1. These data suggest, but do not prove, that FOXM1 restoration of CDK1is sufficient to promote cell cycle progression in TRPM2 KO cells treated with doxorubicin during our experimental time frame.

### TRPM2 deletion increases DNA damage after doxorubicin treatment and reduces expression of DNA repair proteins

**Figure 6:**
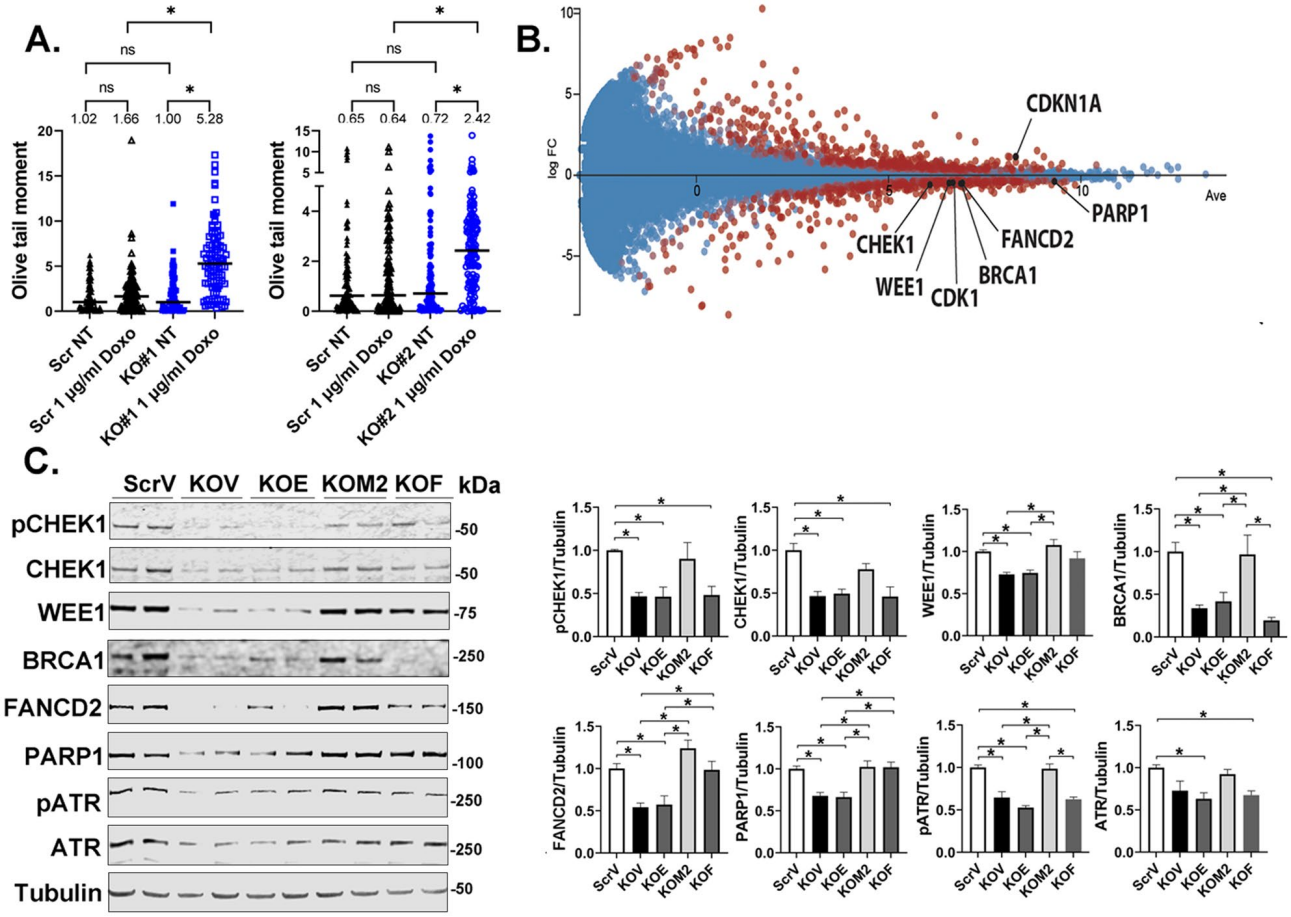
TRPM2 KO increases DNA damage after doxorubicin. (**A**) DNA damage was quantitated with neutral Comet Assay using two Scr and two SH-SY5Y clones with TRPM2 deletion without treatment (NT) or 24 h following treatment with 1 µg/ml doxorubicin. Tails (n = 87–125/group) were analyzed for differences between no treatment and doxorubicin treatment, and Scr compared to KO. Each clone was studied in two experiments with similar results and one experiment is shown for each KO. Figures were prepared using the CometScore 2.0 softwear. Statistics: two-tailed Mann–Whitney test, *p < 0.05. (**B**) RNA seq analysis of expression of DNA damage response genes in TRPM2 KO vs Scr cells after 24 h treatment with doxorubicin. MA plot (log ratio vs abundance) of the RNA seq data of Scr vs KO. Two biological replicas of two clones are combined. Genes with q-value < 0.05 are displayed in red and relevant genes are labeled. Log FC < 0 indicates Scr > KO. Figure was prepared using the Degust 4.1.1 web-tool for RNA seq analysis (https://degust.erc.monash.edu/). (**C**) Western blots of lysates from scrambled control cells transfected with empty vector (ScrV), cells with TRPM2 deletion transfected with empty vector (KOV), E960D mutant (KOE), TRPM2 (KOM2) or FOXM1 (KOF) treated with doxorubicin. Blots were probed with antibodies to DNA damage response proteins including phosphorylated (p) CHEK1, CHEK1, WEE1, BRCA1, FANCD2, PARP1, pATR, ATR, and tubulin. Samples (ScrV, KOV, KOE, KOM2, KOF) were derived from the same experiment and always processed in parallel. Blots from a representative of three experiments are shown. The different probes for each antibody are made explicit with white space and delineated with a black box. Densitometry measurements from three Western blots for each protein were standardized to tubulin and to each experiment’s scrambled control, and means ± S.E.M. of the three are shown (n = 6). Statistics: unpaired t test, *p < 0.05. Full length gels for Western blots are shown in Supplementary Fig. S5.

ROS, key determinants of DNA damage, are significantly increased in neuroblastoma^25,32^ and myeloid leukemia cells^3^ with TRPM2 deletion treated with doxorubicin, compared to control, from both increased ROS production and reduced antioxidant defenses^3,25,32^. Because DNA damage can halt cell cycle or result in apoptosis if extensive, DNA damage was measured by the Comet tail assay in untreated and doxorubicin treated neuroblastoma cells. Here, the amount of DNA damage was similar between untreated control and cells with TRPM2 deletion (Fig. 6A). Doxorubicin treatment significantly enhanced DNA damage (longer Comet tails) in TRPM2 KO cells but not in scrambled control cells (Fig. 6A, *p < 0.05).

Reduced DNA repair may manifest as enhanced DNA damage. Expression of the DNA repair genes CHEK1, WEE1, BRCA1, FANCD2 and PARP1 was significantly reduced in cells with TRPM2 deletion after doxorubicin treatment, both at the RNA (Fig. 6B) and protein level (Fig. 6C). Phosphorylated and total CHEK1 and phosphorylated protein kinase ATR, involved in sensing DNA damage and activating DNA damage checkpoints, were also reduced in cells with TRPM2 deletion (Fig. 6C). Reconstitution of TRPM2 with wild type but not E960D mutant restored expression of DNA repair proteins and ATR phosphorylation, thereby providing another mechanism (other than modulating expression of cell cycle proteins) by which calcium influx through TRPM2 protects neuroblastoma from doxorubicin injury. Collectively, our data suggest that in cells with TRPM2 deletion treated with doxorubicin, DNA damage is increased through a combination of mechanisms including impaired DNA repair, increased ROS, and down modulation of FOXM1 and E2F1/2, which contribute directly to DNA repair^41–43^. Reconstitution of FOXM1 restored FANCD2, PARP1, and partially WEE1. It is likely that doxorubicin did not cause a significant increase in DNA damage in control Scr cells because ROS levels in those cells are not as high as in cells with TRPM2 deletion after doxorubicin^3,25,32^, DNA repair mechanisms are largely intact, and the Comet Assay used here may not be able to detect the smaller amount of DNA damage that occurred.

### TRPM2 deletion in U937 myeloid leukemia cells also reduced expression of E2F1, FOXM1 and cell cycle and DNA repair proteins

**Figure 7:**
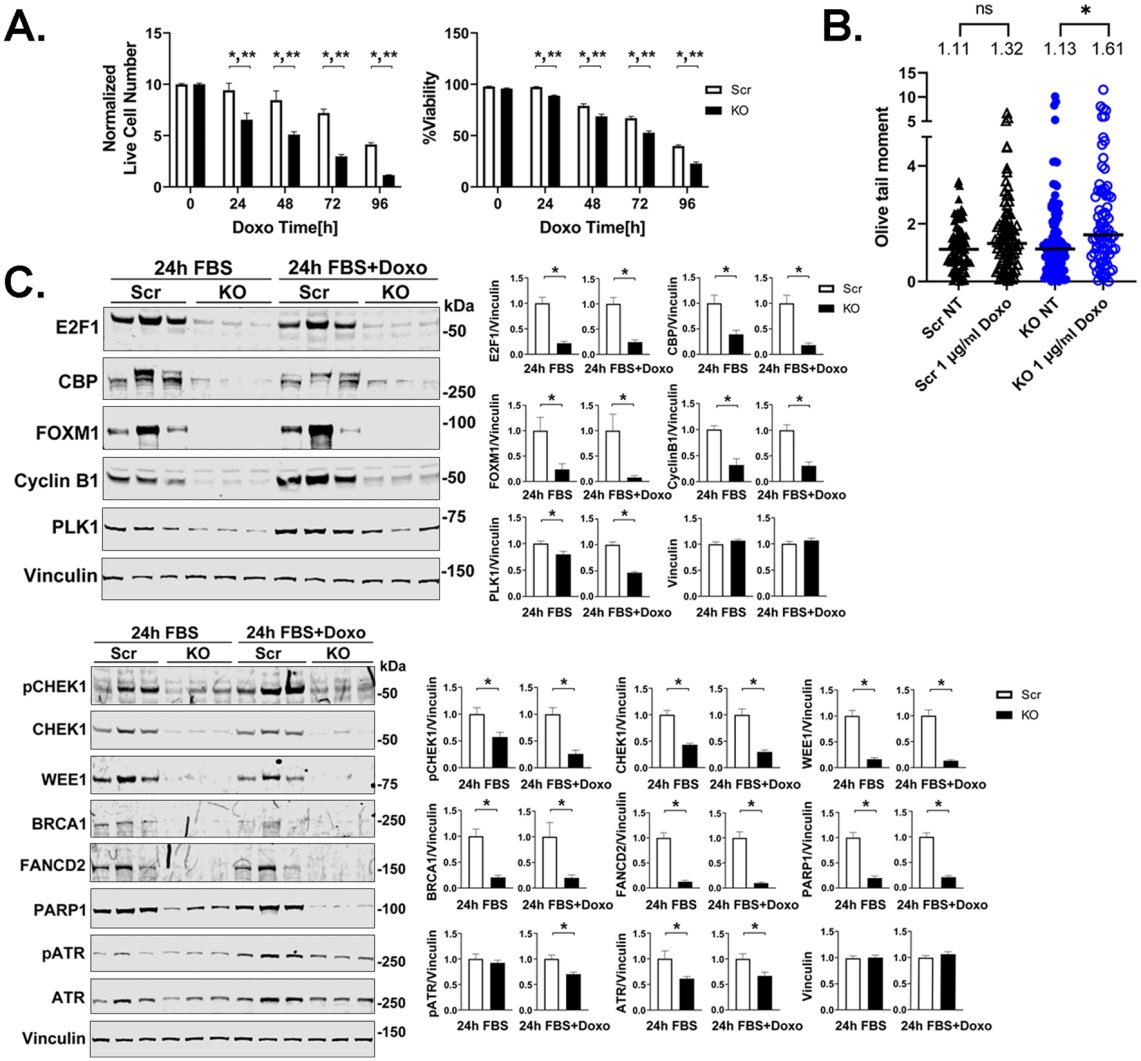
Deletion of TRPM2 in U937 cells. (**A**) TRPM2 KO and Scr U937 cells were plated at time 0, and live cell number and viability (% live cells/total × 100%) measured at 0 to 96 h after treatment with 0.1 µM doxorubicin (n = 7–12/group). Means ± S.E.M. are shown. Statistics: two-way ANOVA, *p < 0.0001, indicates significant differences between Scr vs KO, Group Effect; **p < 0.003 group x doxorubicin exposure time interaction effect (indicates the differences between groups significantly increased with time of doxorubicin exposure). (**B**) DNA damage was quantitated with neutral Comet Assay. Scr (triangles) and KO (circles) U937 cells were studied without treatment (NT), or 24 h following treatment with 1 µg/ml doxorubicin. Open triangles (Scr) and open circles (KO) indicate treatment with doxorubicin. Two experiments were performed with similar results and one is shown. Tails (n = 77–93 /group) were analyzed for differences between no treatment and doxorubicin treatment. Figure was prepared using the CometScore 2.0 softwear. Statistics: two-tailed Mann–Whitney test, *p < 0.05. (**C**) Western blots using lysates from three clones of TRPM2 KO or scrambled control cells were probed with antibodies to cell cycle proteins E2F1, CBP, FOXM1, Cyclin B1, and PLK1 or DNA damage response proteins pCHEK1, CHEK1, WEE1, BRCA1, FANCD2, PARP1, pATR, and ATR. Samples (Scr, KO) were derived from the same experiment and were always processed in parallel. One Western blot for each antibody is shown. Each blot was probed with anti-vinculin antibody as control. The different probes for each antibody are made explicit with white space and each antibody probe is delineated with a black box. Densitometry measurements for three experiments were normalized to vinculin and each blots’ Scr control, and mean ± S.E.M. for each protein from the three (n = 9) is shown. Statistics: unpaired, two tailed t test, *p < 0.02. Full length gels for Western blots are shown in Supplementary Fig. S6.

To begin to generalize our results to other cancers, we studied U937 leukemia cells with TRPM2 deletion^3^. Viability of leukemia cells with TRPM2 deletion was reduced after doxorubicin treatment compared to control and the magnitude of the relative reduction increased with time of doxorubicin exposure (Fig. 7A). DNA damage was significantly increased in U937 cells with TRPM2 deletion treated with doxorubicin compared to untreated cells, but not in Scr control (Fig. 7B). Transcription factors E2F1, FOXM1, CBP and cell cycle proteins Cyclin B1 and PLK1 were reduced in untreated and doxorubicin treated U937 cells with TRPM2 deletion (Fig. 7C). Similar to TRPM2 KO SH-SY5Y cells exposed to doxorubicin treatment, expression of DNA repair proteins was reduced in U937 cells in which TRPM2 was deleted (Fig. 7C).

## Discussion

**Figure 8:**
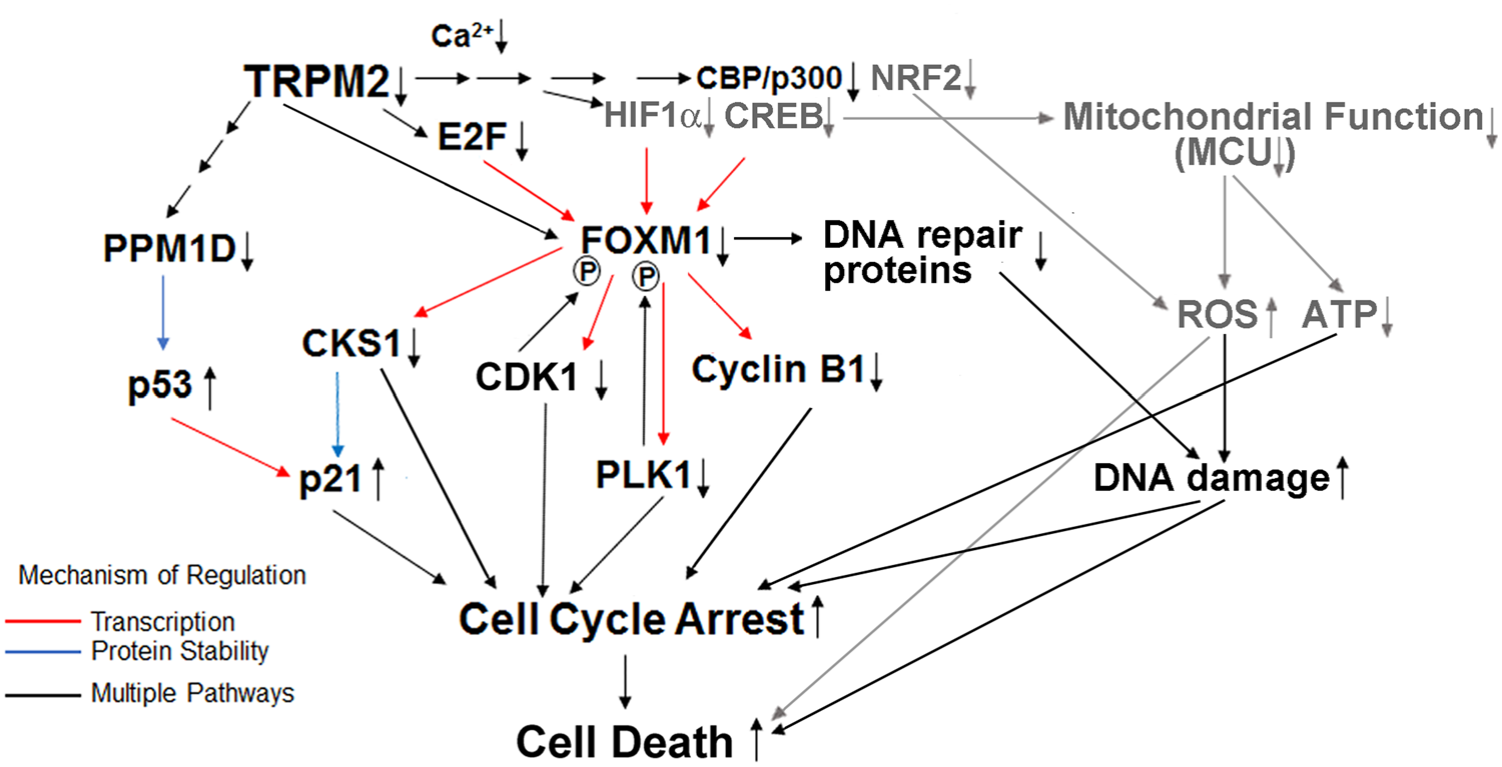
Schema of TRPM2 modulation of cell cycle progression and DNA repair in neuroblastoma. Decreased TRPM2 was previously shown to down modulate HIF-1α, CREB, and NRF2 expression. Previously reported findings are shown here with grey arrows and lettering, and new finding reported here in black. TRPM2 regulates E2F1 and its target FOXM1. FOXM1 regulates downstream cell cycle effectors including Cyclin B1, PLK1, CDK1, and CKS1, which modulates the ubiquitination and degradation of p21. In TRPM2 deletion, E2F1, FOXM1, and downstream targets involved in cell cycle are decreased. p21 is increased, through reduced ubiquitination/degradation by CSKI and transcriptional upregulation by increased p53. Reduced ATP from mitochondrial dysfunction may also reduce the ability of the cell to meet enhanced metabolic demands of the cell cycle. Together these pathways modulate increased cell cycle arrest in TRPM2 depletion. FOXM1 and E2F1 also regulate proteins in the DNA damage response and E2F1 participates directly in DNA repair. In cells with TRPM2 deletion, increased DNA damage results from both increased ROS and reduced DNA repair and contributes to delayed cell cycle progression and increased cell death.

TRPM2 has an important role in cell survival, protecting cells from oxidative stress and ischemic injury^30,52,53^. It is highly expressed in many cancers and in acute myeloid leukemia^20,21,54^, consistent with its role in preserving viability and promoting tumor growth. Previous studies have elucidated the important role of TRPM2 in regulation of mitochondrial function, calcium uptake, bioenergetics, and ROS levels^21,25^. The results of our current studies indicate two additional novel mechanisms by which TRPM2 protects cells from doxorubicin treatment: increased expression of cell cycle proteins and enhanced DNA repair (Fig. 8). Decreased expression of transcription factors E2F1/2, FOXM1, and CBP, and downstream proteins critical for cell cycle progression, including Cyclin B1, CDK1, PLK1, CKS1, B-MYB, and members of the DREAM complex were found in TRPM2 KO cells, particularly after doxorubicin treatment. Furthermore, the key inhibitor cyclin-dependent kinase p21 was significantly increased. Progression of cells through S and G2/M phases of cell cycle was impaired in cells with TRPM2 deletion after doxorubicin treatment. Expression of DNA repair proteins was decreased in cells with TRPM2 deletion compared to control, and DNA damage was increased and may contribute further to reduced cell cycle progression. Downregulation of the same cell cycle and DNA repair regulators was found in U937 myeloid leukemia cells in which TRPM2 was deleted.

The first major finding is that two key transcriptional regulators involved in carcinogenesis, FOXM1 and E2F1, are reduced in cells with TRPM2 deletion compared to control cells^38,55^. FOXM1 is expressed in highly proliferative cells including progenitor cells and regenerating tissue, and both FOXM1 and E2F1 are master regulators in cancer^38–40,56^. FOXM1 has a key role in promoting tumor cell proliferation, cell cycle progression, DNA damage repair, angiogenesis, and drug resistance, and elevated FOXM1 correlates with poor prognosis in many cancers^38,39,57^. FOXM1 downregulation is important therapeutically because it has many transcriptional targets. It strongly activates promoters of genes involved in G2/M phase and weakly those in S^58^. FOXM1 inhibition results in decreased proliferation, EMT and migration, metastasis, and DNA repair^35,41^. FOXM1 is a major driver of embryonic stem cell regulatory programs in neuroblastoma patients with MYCN amplified tumors and stage 4 non-amplified tumors, and expression correlates with poor patient outcome and therapy resistance^56^. In these tumors, FOXM1 controls a large set of genes involved in cell cycle control and DNA damage response. Our data suggest that targeting TRPM2 may be a novel approach to reduce FOXM1 expression and increase doxorubicin sensitivity in neuroblastoma and other malignancies.

The decrease in FOXM1 in cells with TRPM2 deletion may result from a number of mechanisms. Transcription factors which bind to the FOXM1 promoter and positively regulate its transcription include CREB, HIF-1α, c-myc, E2Fs, and CBP/p300^38^. Reduced CREB, HIF-1α, and c-myc were previously demonstrated in TRPM2 KO cells^3,21,25,26^ and reduced E2F1/2 on a transcriptional basis is demonstrated here. CBP serves as coactivator for many transcription factors including E2Fs and CREB^46,47^; its reduced expression in TRPM2 KO cells may contribute to reduced FOXM1 transcription and expression^46^. In addition, the transcription factor p53 is a repressor of FOXM1 transcription^59^, and p53 is increased in TRPM2 depletion^60^, contributing to reduced FOXM1. FOXM1 expression and activity may also be affected by translational or posttranslational mechanisms^38,39^. For example, FOXM1 is acetylated by CBP/p300 during S phase and G2/M, and reduced expression of CBP in TRPM2 KO may impair FOXM1 functional activity through decreased FOXM1 acetylation^61^.

Cyclin B1, CDK1, PLK1, and CKS1 facilitate cell cycle progression into S and M phase, and are transcriptional targets of FOXM1^48,49^. Another major finding is that they are significantly decreased in cells with TRPM2 deletion after doxorubicin, playing important roles in reduced cell cycle progression to S and G2/M. CHIP analysis confirmed the role of FOXM1 in their reduced transcription. Cyclin B1 is a regulatory protein expressed predominantly in G2/M phase of the cell cycle, which binds to CDK1 to promote mitosis. Cyclin B1 is phosphorylated by PLK1 and CDK1, promoting cyclin B1 relocation to the nucleus where it is active^38^. CDK1 and PLK1 are important for phosphorylation and activation of FOXM1, the cyclin B1/CDK1 complex, and other targets in S and G2/M progression^38,62^. Activation of FOXM1 is also dependent on the MMB/DREAM Complex. Components of activator DREAM complexes are reduced in TRPM2 KO including LIN37 and B-MYB, which are required for FOXM1 target gene promoter binding and contribute to decreased expression of G2/M genes^35,63^.

CKS1, cyclin-dependent regulatory subunit 1, has a number of roles in cell cycle progression and DNA damage responses including facilitation of ubiquitination and degradation of cyclin-dependent kinase inhibitors p21 and p27^50^. Our data show p21 expression is significantly increased in neuroblastoma cells with TRPM2 deletion, due to both reduced degradation initiated by CKS1 and increased p53-mediated transcription. PPM1D (protein phosphatase Mg^2+^/Mn^2+^ dependent 1D, Wip1) dephosphorylates p53, inhibiting its activity, and facilitates p53 ubiquitination and degradation; its decrease in TRPM2 deleted cells (Supplementary Fig. S7) may contribute to the increase in p53 expression and function (Figs. 3, 8). Increased expression of p21 inhibits cyclin-CDK1, -CDK2, and -CDK4/6 activity, stabilizes and activates RB proteins, and inhibits cell cycle progression at G1/S and G2/M^64^.

Mitochondrial function is significantly decreased in cells with TRPM2 deletion, evidenced by reduced mitochondrial calcium uptake, oxygen consumption rate, and ATP production^3,21,25^. Expression of the mitochondrial calcium uniporter (MCU) and peak mitochondrial calcium uniporter current is significantly reduced in the TRPM2 KO^26,30^, mediated partially by reduced CREB. Reduced MCU expression and MCU activity^21^ contribute to reduced mitochondrial calcium uptake in cells with TRPM2 deletion, which is required for ATP production^65^. Cells in G1/S have the highest mitochondrial ATP output, and an increase in ATP is essential for G1/S transition^66^. Decreased expression and activity of MCU in neuroblastoma cells with TRPM2 deletion may contribute to reduced cell cycle progression during G1/S and G2/M through inability to meet increased energy requirements^67^.

Doxorubicin is known to affect cell cycle progression through generation of ROS which damage DNA and membranes, and through disruption of topoisomerase II mediated DNA repair. A major finding here is that DNA damage is significantly increased in neuroblastoma and AML cells with TRPM2 deletion after exposure to doxorubicin compared to control, in agreement with results in breast adenocarcinoma^68^. DNA damage is enhanced in cells with TRPM2 deletion after doxorubicin treatment through several mechanisms including significantly increased ROS^3,25^, decreased antioxidants^32^, and decreased FOXM1, E2F1, and DNA repair proteins, reported here. E2F1 regulates both expression of DNA repair genes and directly functions in DNA end resection, recruiting and retaining DNA repair factors at sites of double stranded DNA breaks and in DNA end processing^37,42^. FOXM1 and PLK1 regulate expression and/or function of a number of DNA damage checkpoint and repair proteins^69^. WEE1, CHEK1, BRCA1, FANCD2, PARP1, and phosphorylation of CHEK1 and ATR were significantly reduced in TRPM2 deleted neuroblastoma and myeloid leukemia cells after doxorubicin treatment. DNA damage can delay cell cycle progression to allow time for DNA repair, and this together with the increase in p21 and decline in CDK1 in cells with TRPM2 deletion contribute to cell cycle delay observed here. In addition, PLK1 has been shown to be essential for cell cycle restart post the pause for DNA repair, and through its reduction in TRPM2 deletion may also contribute to cell cycle delay^69^.

Reconstitution experiments here demonstrated that wild type TRPM2 but not the calcium pore mutant E960D restored cell proliferation, viability, and cell cycle and DNA repair protein expression in cells with TRPM2 deletion, underscoring the key role of calcium entry through TRPM2. In neuroblastoma and other tumors, the importance of calcium entry in cell proliferation, cell cycle progression, cell cycle checkpoint control, and resistance to death has been reviewed recently^66,70,71^. Calcineurin and calcium-calmodulin dependent protein kinases regulate key transcription factors and many cell cycle proteins involved in cell cycle progression. The impact of changes in calcium levels on G1 and S phase as well as on G2/M and metaphase to anaphase transition has been demonstrated^66^, and now shown here to include TRPM2 activation.

Our results demonstrate that TRPM2 promotes cell cycle progression, viability, and DNA repair in neuroblastoma and myeloid leukemia after doxorubicin treatment (Fig. 8). When TRPM2 is deleted, key transcription factors FOXM1 and E2F1, which modulate expression of cell cycle and DNA repair proteins, are reduced, particularly after doxorubicin. An inhibitor of the cell cycle p21 is increased. Reduced MCU and mitochondrial Ca^2+^ entry severely impair mitochondrial function, reducing ATP production and preventing the cell from meeting enhanced metabolic demands needed during cell cycle, as well as increasing pathologic ROS production. ROS and subsequent DNA damage are increased and DNA repair proteins are reduced, contributing further to cell cycle delay. Together, these results confirm the potential role of TRPM2 as a therapeutic target through pathways including reducing FOXM1 and E2F1 expression, which impairs cell cycle progression, DNA repair, and increases doxorubicin sensitivity.

## Materials and methods

### Cell culture of neuroblastoma and leukemia cell lines

The neuroblastoma cell line SH-SY5Y and the myeloid leukemia cell line U937 were obtained from the American Type Culture Collection (ATCC, Manassas, Va, USA). SH-SY5Y cells were cultured in 50% DMEM and 50% Ham’s F-12 (DMEM/F-12 50/50) media supplemented with 10% heat-inactivated FBS^21,25^. U937 cells were cultured in RPMI 1640 with 10% FBS^3^.

### Deletion of TRPM2 with CRISPR and generation of stably transfected neuroblastoma and leukemia cell lines

TRPM2 knockout (KO) and scrambled control SH-SY5Y neuroblastoma cells^25^ and U937 myeloid leukemia cells^3^ were generated in the Miller Laboratory with CRISPR technology as described previously and authenticated with RT-PCR and Western blotting. In these cells, TRPM2 genomic DNA encoding the first 40 amino acids were deleted and the remaining TRPM2 sequence was frameshifted. Scrambled (Scr) control cells used in experiments went through the CRISPR protocol except that they were exposed to scrambled gRNA instead of TRPM2 targeted. Engineering and authentication of the calcium-impermeant TRPM2 E960D mutant are as described^25,26^. In TRPM2 reconstitution experiments, SH-SY5Y KO cells were transfected with wild type TRPM2, TRPM2 E960D mutant^31,51^, or empty vector using the Neon Transfection System as described^32^.

FOXM1 has two transcriptionally active isoforms, FOXM1b and FOXM1c. The FOXM1b isoform was selected for reconstitution experiments because it is overexpressed in many cancers and has greater transforming potential than FOXM1c^72^. The FOXM1b construct pCW57.1 was obtained from Addgene (catalog number 68811, Watertown, MA). It was subcloned into pcDNA3.1V5/His plasmid (Invitrogen), by first amplifying the FOXM1b sequence using two primers: FOXM1 BAMH1 F: 5′ GCTCGGATCCACATGAAAACTAGCCCCCGTCGG and FOXM1 NOT1 R: 5′ CTCGAGCGGCCGCTGTAGCTCAGGAATAAACTG containing cutting sites for two restriction enzymes, BAMH1 and NOT1. The amplified fragment and pcDNA3.1V5/His vector were then cut at BAMH1 and NOT1 sites, ligated into pcDNA3.1V5/His, and used for transfection as described for TRPM2. Clones were confirmed by sequencing.

### Cell proliferation assay and apoptosis analysis

Cell proliferation and viability were assessed by XTT or by cell counting with trypan blue exclusion^32^. In some experiments, cells were treated with doxorubicin (0.3 μM for SH-SY5Y cells, 0.1 µM for U937; Fresenius, Kabi USA, LLC, Lake Zurich, IL). Apoptosis was quantitated by staining with BD Annexin V Apoptosis Detection Kit I and analysis using Flow cytometry on 10-color BD FACSCanto.

### Xenograft tumors with TRPM2 deletion

Xenograft tumors were generated using TRPM2 KO, scrambled control SH-SY5Y cells, or KO cells reconstituted with wild type TRPM2 as described previously^25^. Athymic Nude-FOXn1^nu^ female mice (Harlan Laboratories, Inc., Indianapolis, IN) were injected in one flank with 1.5 × 10^7^ SH-SY5Y cells in which TRPM2 was deleted with CRISPR, cells in which the deletion was reconstituted with TRPM2, or scrambled CRISPR control cells. Approximately 10–12 mice per group were used in each of two experiments, and tumor volume was measured over 10 weeks with a calipher as described^25^. All procedures and protocols used in this study were approved by the Institutional Animal Care and Use Committee of the Pennsylvania State University College of Medicine (PSCOM) and conformed to guidelines and regulations. Studies were in compliance with ARRIVE guidelines.

### RNA-seq

RNA from TRPM2 KO or scrambled control SH-SY5Y cells treated with and without doxorubicin was prepared using RNeasy kit (Qiagen, Hilden, Germany) and analyzed by the PSCOM Genomic Science Core Facility. Differential expression analysis between conditions (two biological replicates per clone, two clones per condition) was performed using the EdgeR package. The resulting P values were adjusted using Benjamini–Hochberg to control the false discovery rate (FDR or q-value). Genes with an adjusted P-value (FDR or q-value) < 0.05 found by EdgeR were assigned as differentially expressed. Figures were prepared using the Degust 4.1.1 web-tool for RNA seq analysis (https://degust.erc.monash.edu/). The RNA seq data for untreated and doxorubicin treated SH-SY5Y cells discussed in this publication are deposited in NCBI’s Gene Expression Omnibus^44,45^ and are accessible through GEO Series accession number GSE197243 (https://www.ncbi.nlm.nih.gov/geo/query/acc.cgi?acc=GSE197243).

### Immunoblot analysis

Western blotting was performed as described previously^25^. Blots were probed with the antibodies: Abcam, Cambridge, MA—anti-B-MYB (1:1000), anti-CDK1 (1:500), anti-CSK1 (1:500), anti-DP1 (1:500), anti-E2F2 (1:1000), anti-p130 (1:500); Bethyl Laboratories, Montgomery, TX—anti-TRPM2-C (1:300); Cell Signaling Technology INC, Boston, MA—anti-ATR (1:1000), anti-pATR (1:1000), anti-BRCA1 (1:1000), anti-CBP (1:5000), anti-CHEK1 (1:1000), anti-pCHEK1 (1:1000), anti-Cyclin B1 (1:1000), anti-E2F1 (1:1000), anti-FANCD2 (1:1000), anti-FOXM1 (1:1000), anti-GAPDH (1:10,000), anti-lamin (1:1000) anti-Lin37 (1:500), anti-PARP1 (1:1000), anti-PLK1 (1:1000), anti-p21 (1:1000), anti-p53 (1:500), anti-pRB (1:1000), anti-RB (1:1000), anti-vinculin (1:5000), anti-WEE1 (1:1000); Invitrogen, Carlsbad, CA—anti-V5-HRP (1:2000); Sigma, St. Louis, MO—anti-tubulin (1:10,000). Secondary antibodies were conjugated to IRDye 800CW or IRDye 680RD (donkey anti-rabbit, 1:20,000, or donkey anti-mouse, 1:20,000) and bands quanitated with the Odyssey CLx fluorescence scanner. All bands were analyzed with Image Studio. Samples (Scr, KO) were derived from the same experiment and were always processed in parallel on a blot. Each blot was probed individually with an antibody, then reprobed with additional single antibodies or two at the same time when molecular weights were sufficiently different (> 30 kDa). The different probes for each antibody were made explicit in the figures with white space and delineated with a black box. For each protein, at least three experiments were performed, except in Fig. 5C where 4–5 experiments were performed. For each figure, subgroups shown (A, B, etc.) were from the same experiment unless the high number of antibodies made additional probing not feasible. Blots were cut to maintain 6 band widths above and below the band space permitting.

### Cell cycle analysis

Scrambled control and TRPM2 KO SH-SY5Y cells were synchronized by serum starvation for 12–18 h, then cultured for 24 h in 10% FBS with or without treatment with 0.3 µM Doxorubicin. Cells were stained using Invitrogen FxCycle PI/RNase Staining solution and analyzed by flow cytometry on BD Accuri C6 flow cytometer.

### Comet assay to measure DNA breaks

DNA damage was evaluated using the TREVIGEN Comet assay kit (4250-050-K) neutral assay. Nikon Eclipse TE2000-U was used for imaging and percent tail DNA was calculated using CometScore 2.0 software. Medians ± standard error are shown. Statistical analysis was performed using two-tailed Mann–Whitney test. Figures were prepared using the CometScore 2.0 softwear (http://rexhoover.com/index.php?id=cometscore.

### Chromatin immunoprecipitation assay

CHIP analysis of FOXM1 binding to Cyclin B1, CDK1, PLK1 and CKS1 promoters was performed with KO or Scr control SH-SY5Y cells, synchronized by serum deprivation, followed by refeeding with media containing 10% FBS and 0.3 µM doxorubicin for 24 h. The procedure was based on a protocol from Dr. Richard Myer’s lab^73^. Purified CHIP DNA was used for qPCR based on previously described primers: Cyclin B1 Promoter^74^; CDK1 promoter^74^; PLK1 promoter^74^; CKS1 promoter^48^. qPCR primer localization for amplifying FOXM1 binding area (TS, transcription start site) or random upstream fragment for nonspecific control are shown above the graphs in Fig. 2C.

### RT-qPCR for E2F1, E2F2, CBP, FOXM1, Cyclin B1, CDK1, PLK1, CKS1, and p21

SH-SY5Y KO or Scr control cells were synchronized by serum deprivation for 12–18 h, then refed with media containing 10% FBS with or without 0.3 µM doxorubicin for 24 h. RNA was prepared from these cells using RNeasy kit (Qiagen, Hilden, Germany). First strand cDNA synthesis was performed using Super Script kit (Invitrogen by Life Technologies). Quantitative real-time PCR reaction was performed using first strand cDNA reaction, Quantabio (Beverly, MA) PerfectCT SybR Green Fastmix ROX and primers for E2F1, E2F2, CBP, FOXM1, Cyclin B1, CDK1, PLK1, CKS1, and p21 described previously^75^. Ribosomal protein Rpl32 was used as a reference gene^76^. The PCR results were analyzed as relative mRNA level of cycle threshold (CT) value normalized to the scrambled CRISPR/cas9 neuroblastoma cells in each group for each experiment as control.

### Statistical analysis

Results are expressed as mean ± S.E.M. or median ± S.E.M. (Comet Assay). For analysis of cell growth, xenograft tumor growth and apoptosis analysis, two-way ANOVA was used. Only when statistical significance was detected across groups was sub-analysis between any two groups performed with two-way ANOVA. Results were analyzed with two-way ANOVA for differences between groups (Group Effect), increase in differences between groups with time, and increase in the differences between groups with time of doxorubicin exposure). For other analysis one-way ANOVA, T-test, or two-tailed Mann–Whitney test was used as specified in Figure Legend and p ≤ 0.05 was taken to be statistically significant.

## Supplementary Information

Supplementary Figures.

## Data Availability

RNA seq data have been deposited in NCBI’s Gene Expression Omnibus and are accessible through GEO Series accession number GSE197243. Other data generated during and/or analyzed in the current study are available from the corresponding author on reasonable request.
