# Supplementary material for: The human ion channel TRPM2 modulates cell survival in neuroblastoma through E2F1 and FOXM1
Source: Sci Rep. 2022 Apr 15;12:6311. doi: 10.1038/s41598-022-10385-8 (PMC9012789; doi:10.1038/s41598-022-10385-8)
Supplement: Supplementary file 1 — Supplementary Figures. [file 41598_2022_10385_MOESM1_ESM.pdf]

Supplemental Information File

The human ion channel TRPM2 modulates cell survival in neuroblastoma through E2F1 and FOXM1

Iwona Hirschler-Laszkiewicz[1], Fernanda Festa[1,3], Suming Huang[1,2], George-Lucian Moldovan[3], Claudia Nicolae[3], Ashna Dhoonmoon[3], Lei Bao[1], Kerry Keefer[1], Shu-jen Chen[1], Hong-Gang Wang[1,2], Joseph Y. Cheung[4], and Barbara A. Miller[1,3*]

Affiliations: Departments of [1]Pediatrics, [2]Pharmacology, and [3]Biochemistry and Molecular Biology, The Pennsylvania State University College of Medicine, P.O. Box 850, Hershey, Pa 17033, USA

[4]Renal Medicine, Brigham and Women's Hospital, Boston, MA 02115, USA

Supplementary Figure S1

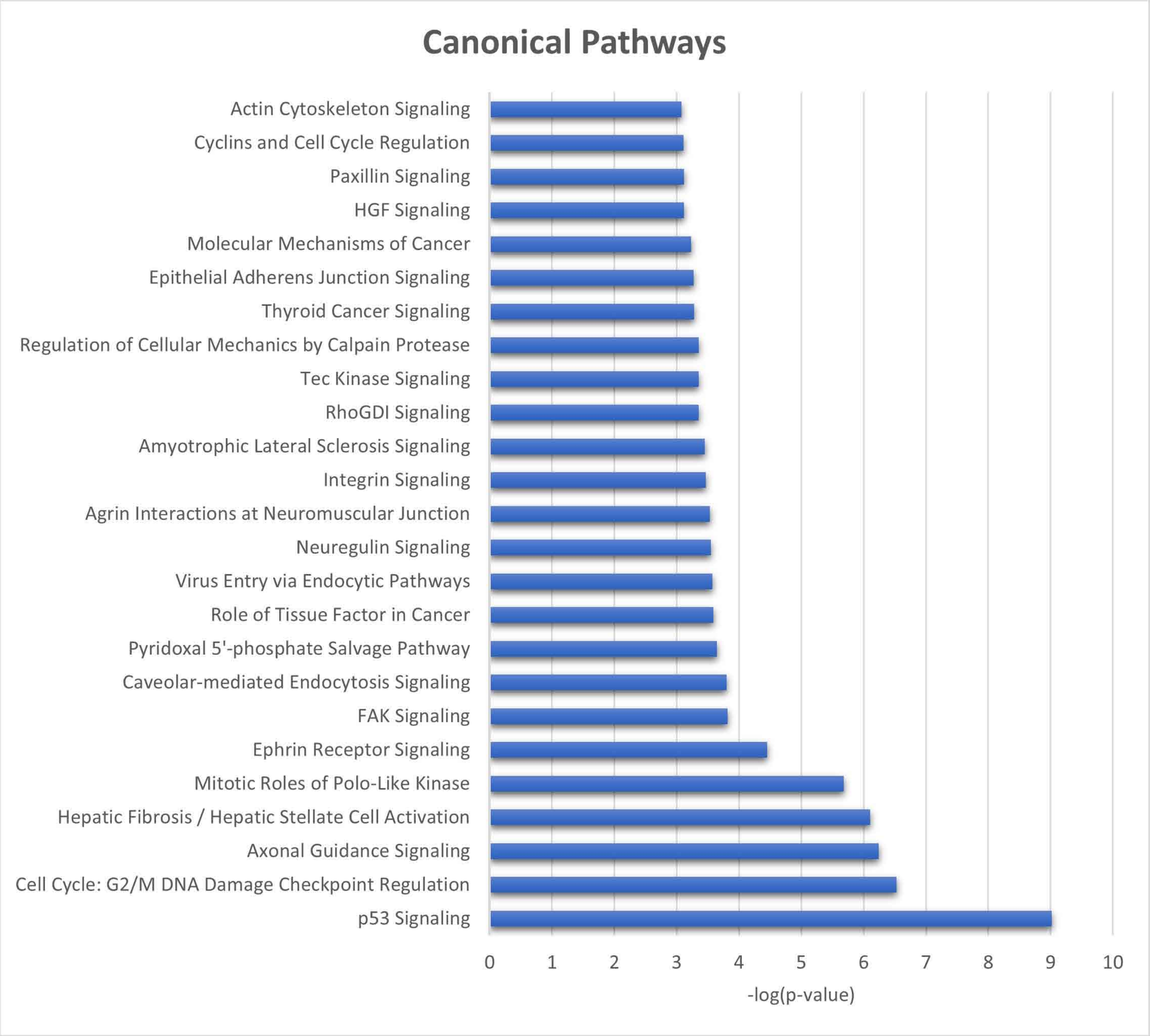

Supplementary Figure S1: Top 25 canonical pathways modulated by TRPM2 deletion in neuroblastoma identified by RNA seq. RNA seq was performed using neuroblastoma SH-SY5Ycells with TRPM2 deletion (TRPM2-KO) and controls (Scr). Cells were treated with 0.3 µM doxorubicin for 24h. For each condition, two clones were tested in duplicate. Genes with q-value <0.05 were considered differentially expressed and the pathway analysis was performed by IPA. The top 25 pathways are displayed in order of increasing statistical significance.

Supplementary Figure S2

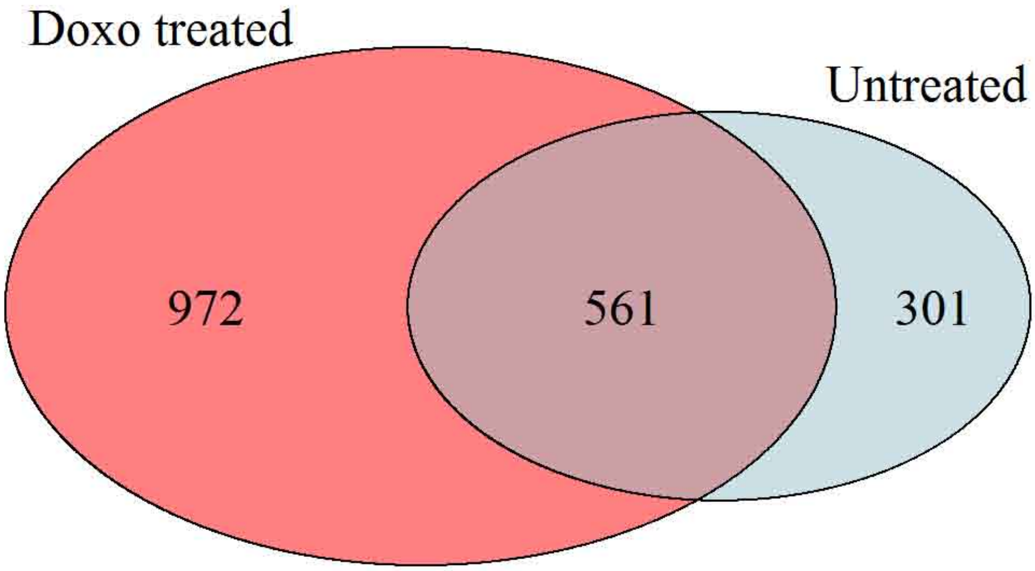

Supplementary Figure S2. RNA seq analysis of genes regulated by TRPM2 in the presence or absence of doxorubicin. Venn diagram shows the number of genes regulated in neuroblastoma cells with TRPM2 depletion (SH-SY5Y TRPM2 KO), with and without doxorubicin treatment, compared to control cells (SH-SY5Y Scr). 561 genes were regulated under both conditions in cells with TRPM2 deletion. Cells were cultured with or without 0.3 uM of doxorubicin for 24 hours. For each condition, two clones from each group were sequenced in duplicate, and the number of genes differentially expressed was calculated.

Supplementary Figure S3

Full gels for Western blot images used to create Figure 3 are shown. Western blots were probed with antibodies to (A) transcription factors E2F1, E2F2, FOXM1, and transcriptional cofactor CBP, (B) DREAM/MuvB complex members and interacting regulatory proteins phospho-RB (pRB), RB, p130, DP1, LIN37, B-MYB, and (C) downstream targets of FOXM1 Cyclin B1, CDK1, PLK1, CKS1, and p21 and p53. Tubulin was used as a loading control. Blots were cut around the expected molecular weights for each protein after probing. Red rectangles mark the bands used.

Full Length Blots Figure 3A

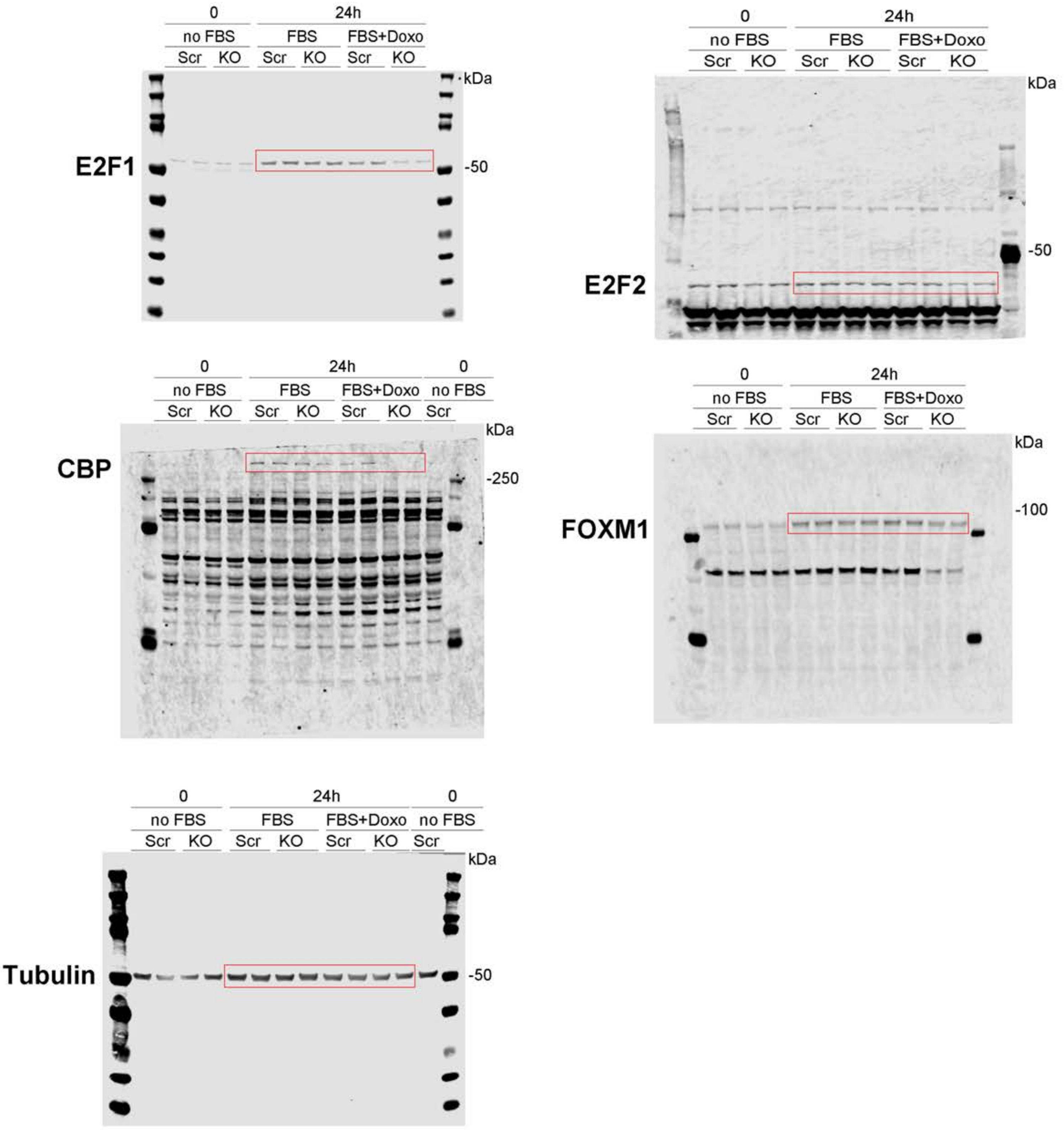

Supplementary Figure S3 continued

Full Length Blots Figure 3B

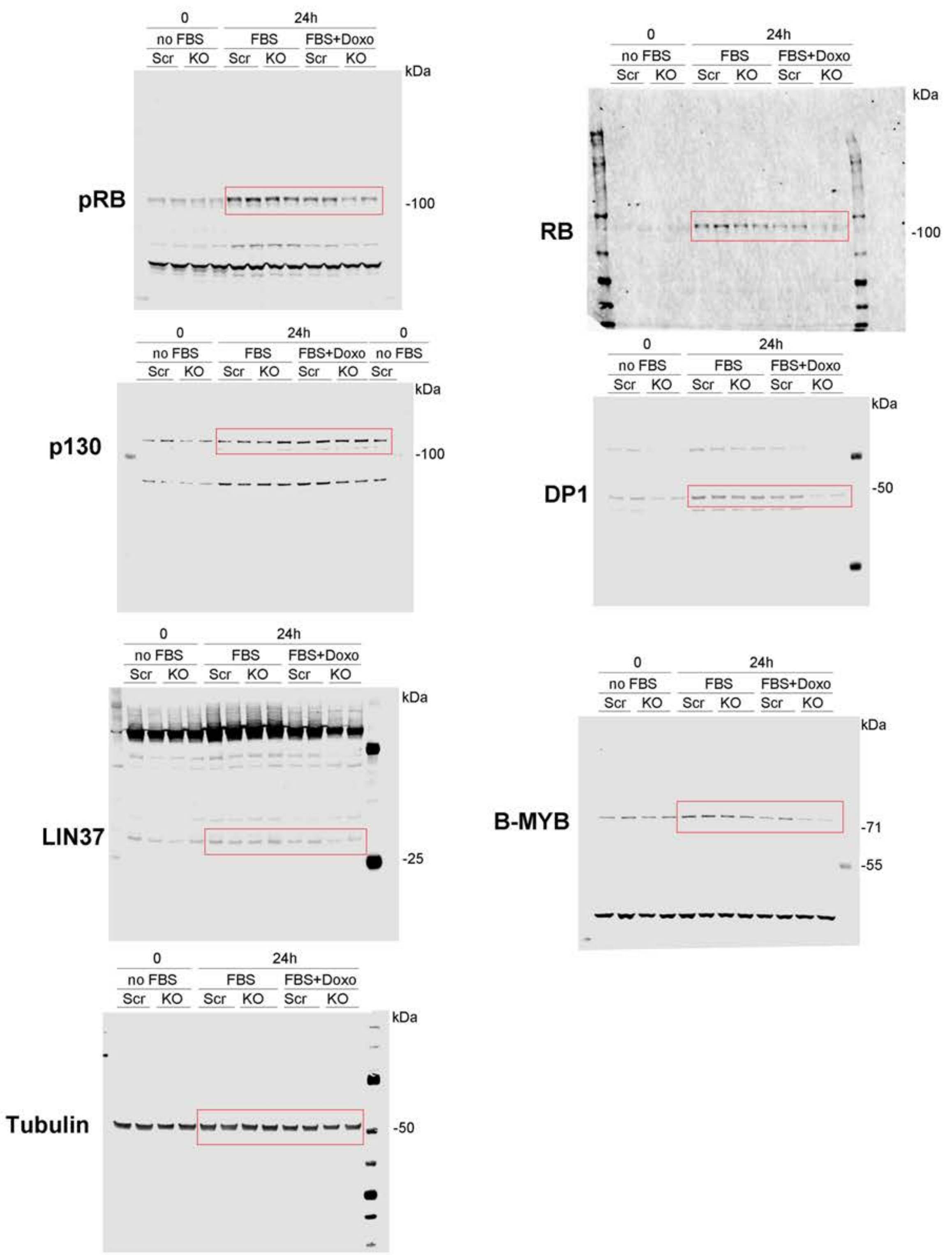

Supplementary Figure S3 continued

Full Length Blots Figure 3C

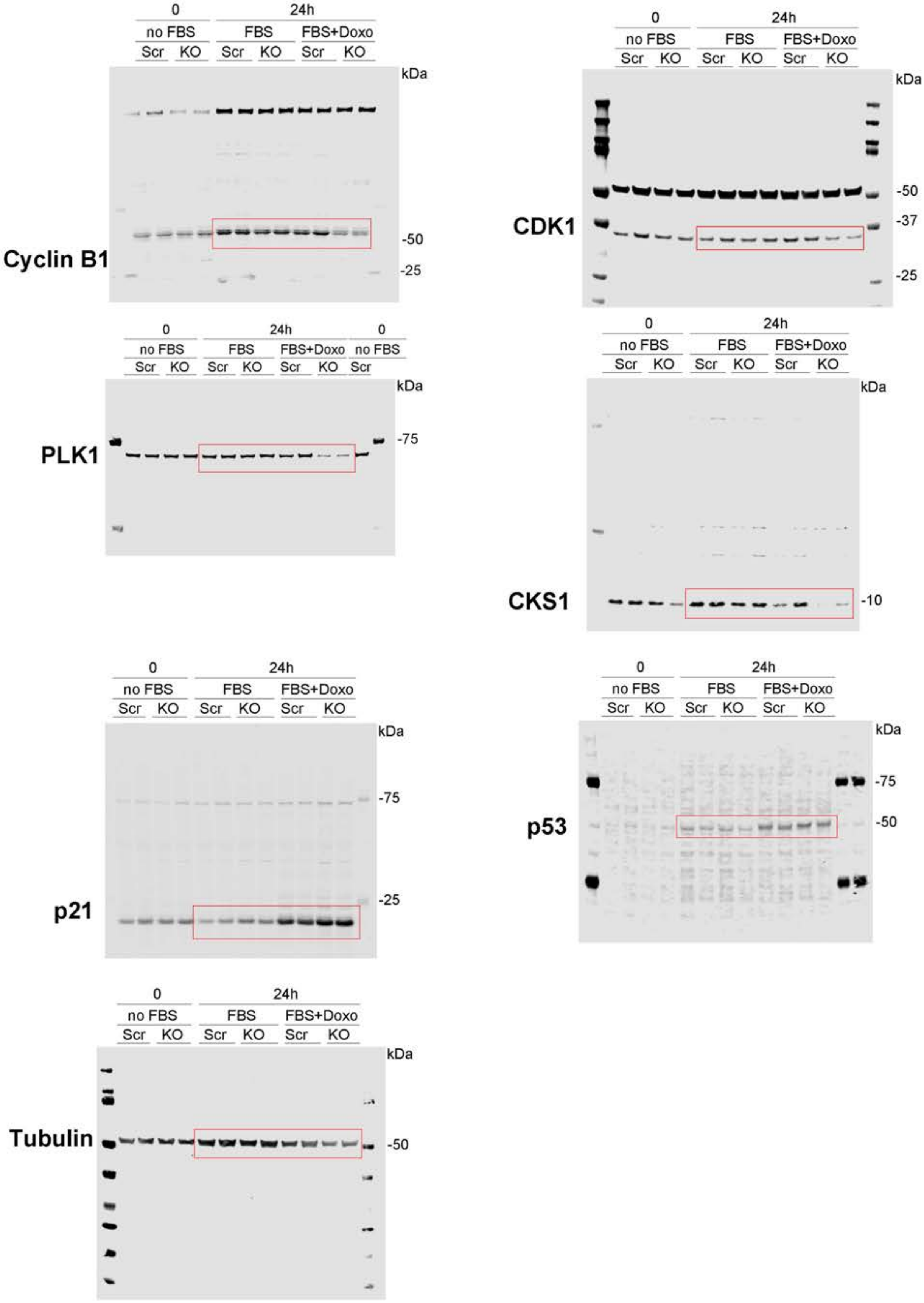

Supplementary Figure S4

Full gels for Western blot images used to create Figure 5C are shown. Western blots were probed with antibodies to V5, E2F1, FOXM1, Cyclin B1, CDK1, PLK1, CKS1, and p21. Tubulin was used as a loading control. Blots were cut around the expected molecular weights for each protein after probing. Red rectangles mark the bands used.

Full Length Blots Figure 5C

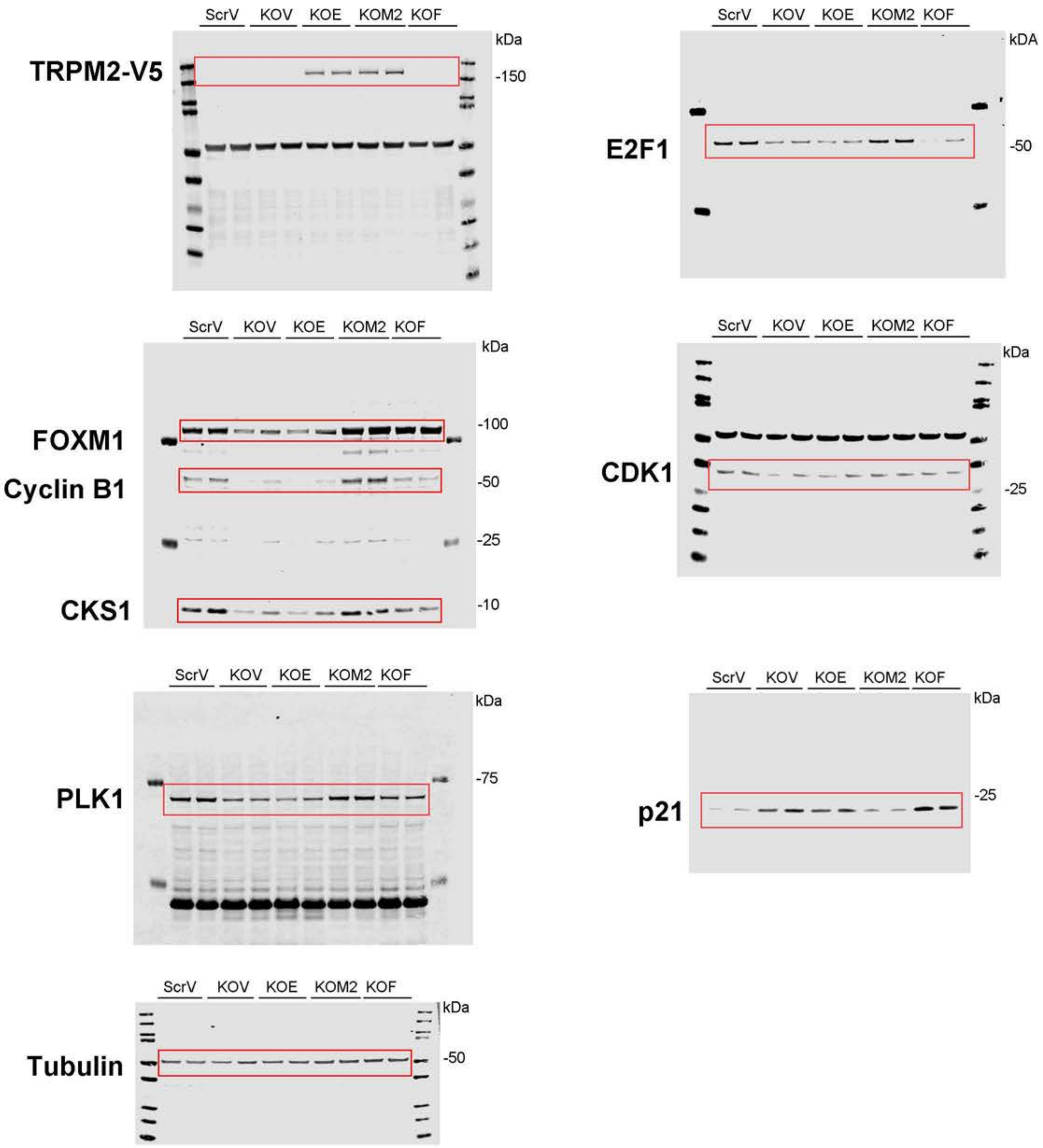

Supplementary Figure S5

Full gels for Western blot images used to create Figure 6C are shown. Western blots were probed with antibodies to pCHEK1, CHEK1, WEE1, BRCA1, FANCD2, PARP1, pATR, and ATR. Tubulin was used as a loading control. Blots were cut around the expected molecular weights for each protein after probing. Red rectangles mark the bands used.

Full Length Blots Figure 6C

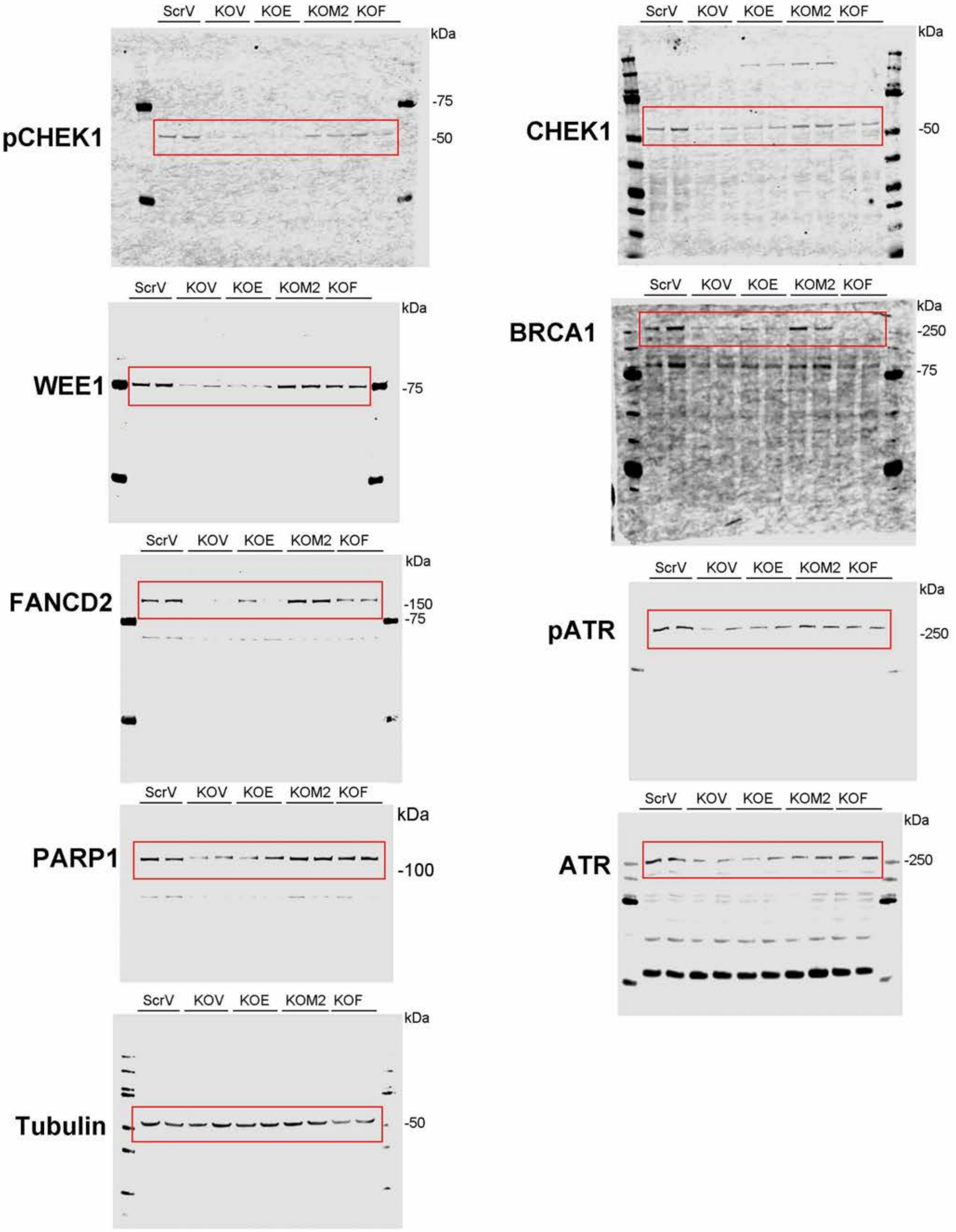

Supplementary Figure S6

Full gels for Western blot images used to create Figure 7C are shown. Western blots were probed in upper gel on Figure 7C with antibodies to E2F1, CBP, FOXM1, Cyclin B1, and PLK1 and in lower gel with antibodies to pCHEK1, CHEK1, WEE1, BRCA1, FANCD2, PARP1, pATR, and ATR. Vinculin was used as a loading control. Blots were cut around the expected molecular weights for each protein after probing. Red rectangles mark the bands used.

Full Length Blots Figure 7C

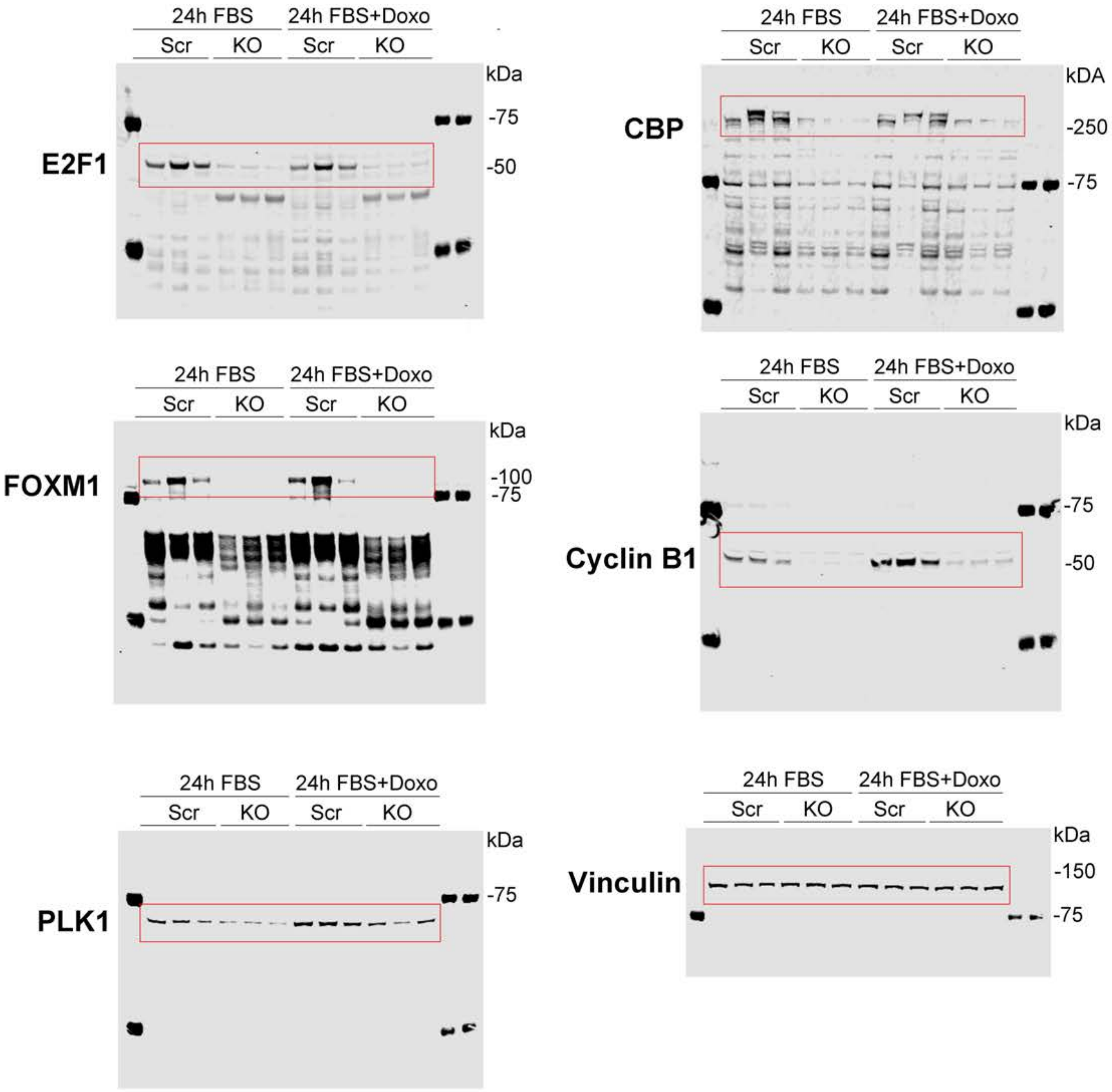

Supplementary Figure S6 continued

Full Length Blots Figure 7C

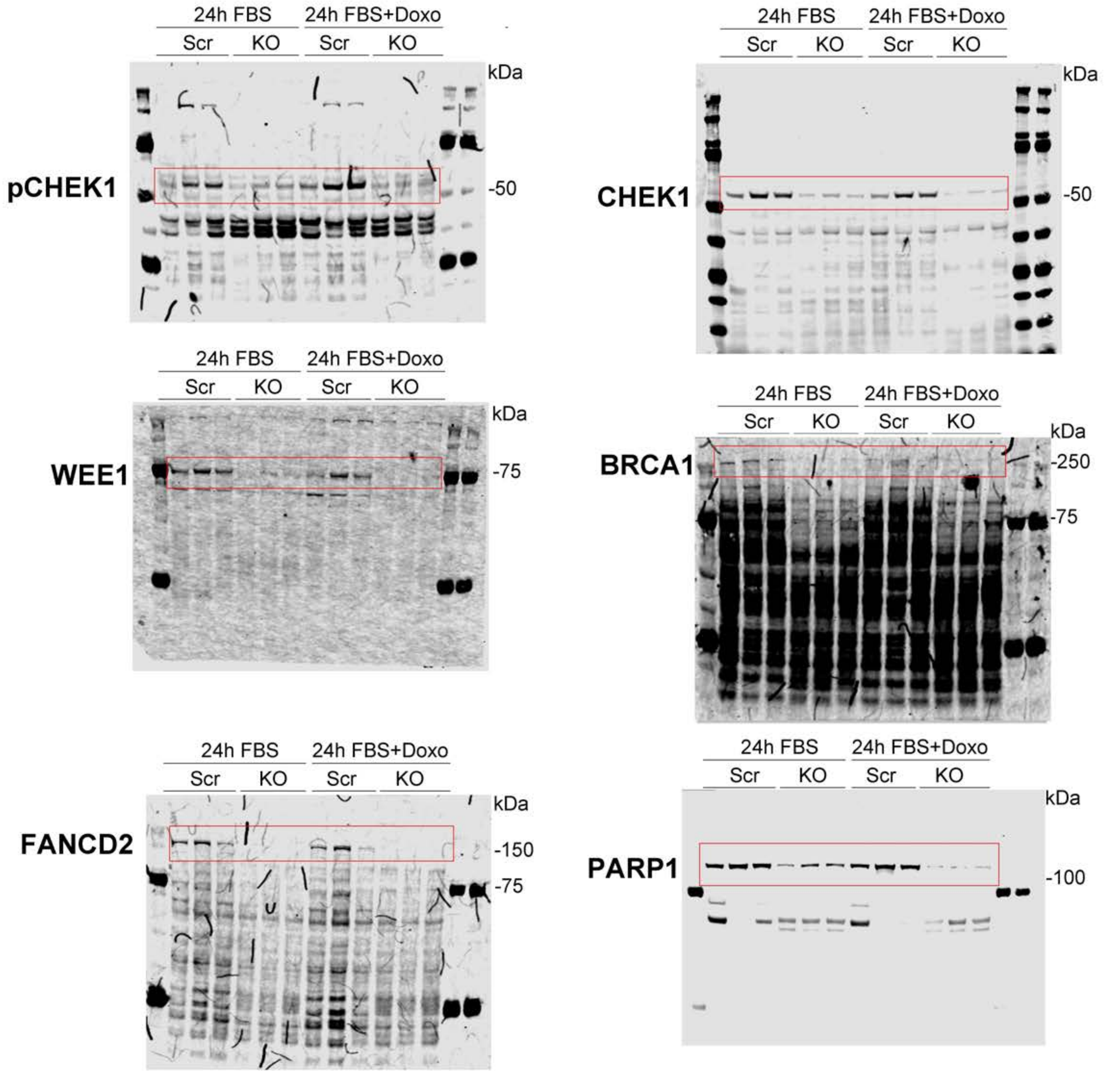

Supplementary Figure S6 continued

Full Length Blots Figure 7C

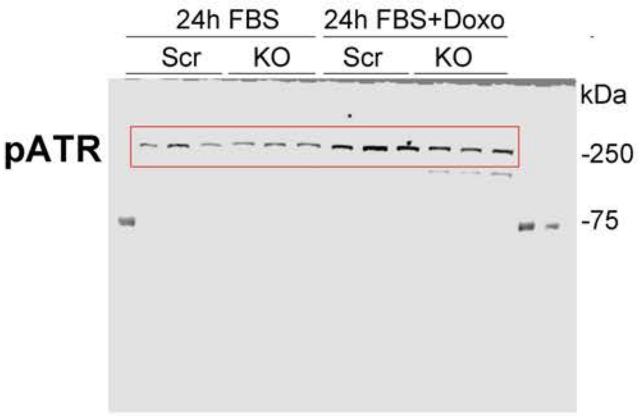

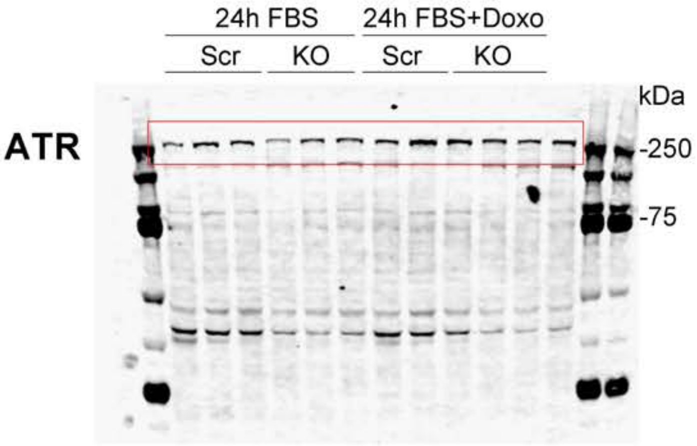

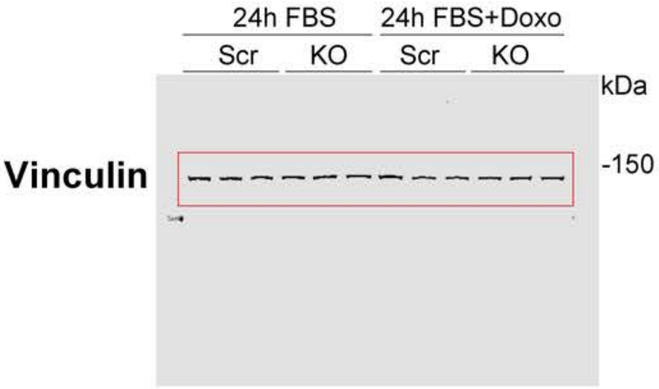

Supplementary Figure S7

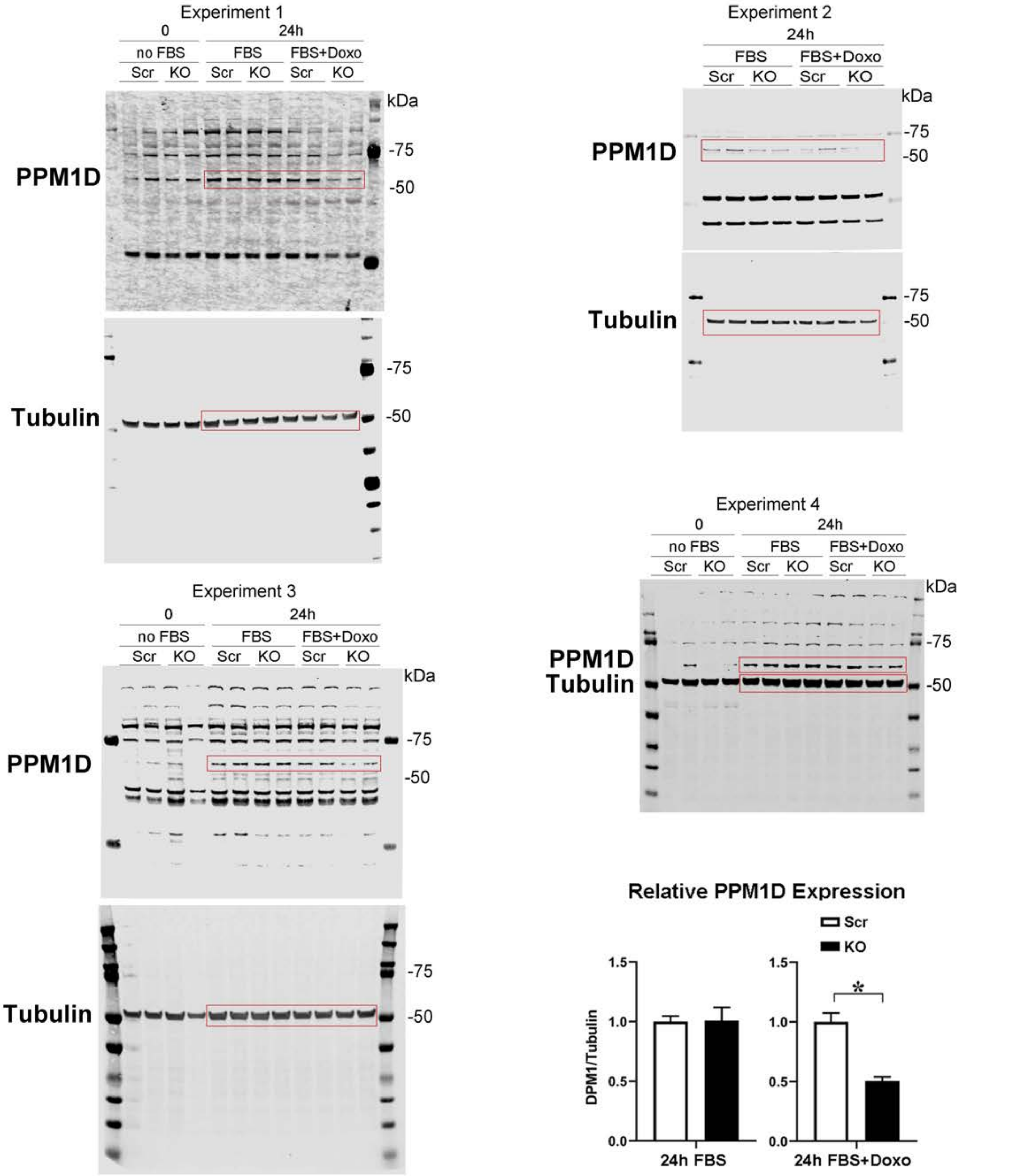

Supplementary Figure S7. PPMID is decreased in SH-SY5Y cells with TRPM2 deletion treated with doxorubicin. Western blotting was performed on two clones of TRPM2 KO and scrambled SH-SY5Y cells after serum deprivation followed by refeeding with 10% FBS with or without 0.3 μM doxorubicin for 24 hours. In four experiments, blots were probed with anti-PPM1D antibody (Abcam 1:1000) and Tubulin. Western blots demonstrated reduced expression of PPM1D in cells with TRPM2 deletion treated with doxorubicin. Samples (Scr, KO) for each blot were derived from the same experiment and always processed in parallel. Full blots are shown with red rectangles marking the bands used. Densitometry measurements from four experiments were standardized to tubulin and each experiment's scrambled control. Means ± S.E.M. for each group (n=8) are shown. Statistics: unpaired t test, *p<0.0001.
